# Whole-Genome Phylogenetic Analysis of Influenza B/Phuket/3073/2013-Like Viruses and Unique Reassortants Detected in Malaysia between 2012 and 2014

**DOI:** 10.1371/journal.pone.0170610

**Published:** 2017-01-27

**Authors:** Xiang Yong Oong, Kim Tien Ng, Joon Ling Tan, Kok Gan Chan, Adeeba Kamarulzaman, Yoke Fun Chan, I-Ching Sam, Kok Keng Tee

**Affiliations:** 1 Department of Medicine, Faculty of Medicine, University of Malaya, Kuala Lumpur, Malaysia; 2 Department of Medical Microbiology, Faculty of Medicine, University of Malaya, Kuala Lumpur, Malaysia; 3 Division of Genetics and Molecular Biology, Institute of Biological Sciences, Faculty of Science, University of Malaya, Kuala Lumpur, Malaysia; Centers for Disease Control and Prevention, UNITED STATES

## Abstract

Reassortment of genetic segments between and within influenza B lineages (Victoria and Yamagata) has been shown to generate novel reassortants with unique genetic characteristics. Based on hemagglutinin (HA) and neuraminidase (NA) genes, recent surveillance study has identified reassortment properties in B/Phuket/3073/2013-like virus, which is currently used in the WHO-recommended influenza vaccine. To understand the potential reassortment patterns for all gene segments, four B/Phuket/3073/2013-like viruses and two unique reassortants (one each from Yamagata and Victoria) detected in Malaysia from 2012–2014 were subjected to whole-genome sequencing. Each gene was phylogenetically classified into lineages, clades and sub-clades. Three B/Phuket/3073/2013-like viruses from Yamagata lineage were found to be intra-clade reassortants, possessing PA and NA genes derived from Stockholm/12-like sub-clade, while the remaining genes from Wisconsin/01-like sub-clade (both sub-clades were within Yamagata Clade 3/Yam-3). However, the other B/Phuket/3073/2013-like virus had NS gene that derived from Stockholm/12-like sub-clade instead of Wisconsin/01-like sub-clade. One inter-clade reassortant had Yamagata Clade 2/Yam-2-derived HA and NP, and its remaining genes were Yam-3-derived. Within Victoria Clade 1/Vic-1 in Victoria lineage, one virus had intra-clade reassortment properties: HA and PB2 from Vic-1B sub-clade, MP and NS from a unique sub-clade “Vic-1C”, and the remaining genes from Vic-1A sub-clade. Although random reassortment event may generate unique reassortants, detailed phylogenetic classification of gene segments showed possible genetic linkage between PA and NA genes in B/Phuket/3073/2013-like viruses, which requires further investigation. Understanding on reassortment patterns in influenza B evolution may contribute to future vaccine design.

## Introduction

Influenza B is a member of the *Orthomyxoviridae* family and has an enveloped structure and segmented negative sense RNA genome [1], and is an important cause of respiratory infections in humans globally. The genome organization of influenza B virus is similar to that of influenza A virus, which consists of eight segments: polymerase basic-1 (PB1), polymerase basic-2 (PB2), polymerase acidic (PA), hemagglutinin (HA), nucleoprotein (NP), neuraminidase (NA), matrix protein (MP), and non-structural protein (NS) [1]. First isolated in 1940 during an epidemic in the USA [2], influenza B viruses have been detected in different geographical regions worldwide [3]. In the 1980s, two antigenically and genetically distinct lineages, B/Victoria/2/1987-like (Victoria lineage) and B/Yamagata/16/88-like (Yamagata lineage), have co-circulated in the human population, based on analysis of the HA gene [4].

Segment reassortment between Victoria and Yamagata lineages based on phylogenetic analysis of the HA and NA genes has been reported since 1999 [5] as a result of unrestricted lineage mixing [6]. An extensive analysis of the whole genome of influenza B viruses in previous studies showed that frequent and complex reassortment of all eight gene segments between and within Victoria and Yamagata lineages continues to play a role in determining the evolutionary changes in both lineages [7–9]. As evident in our recent cross-sectional molecular epidemiological study of influenza B virus, conducted among patients who presented with acute upper respiratory tract infections (URTI) between 2012 and 2014 in Kuala Lumpur, Malaysia [10], it was suggested that the emergence of B/Phuket/3073/2013-like viruses circulating in this country and globally was due to an intra-clade reassortment event within Yamagata Clade 3 (Yam-3), one of the WHO genetic groups within Yamagata lineage [11, 12]. This resulted in the selection of B/Phuket/3073/2013-like virus as a candidate vaccine strain by the WHO for both northern and southern hemispheres for the 2015–2016 influenza season [11, 12].

Previously, the phylogeny of HA and NA genes that we determined allows grouping of influenza B viruses into lineages, WHO genetic groupings (clades) and sub-clades [10], and this permits more detailed classification of inter-clade or intra-clade reassortants. However, these groupings were not extended to other gene segments of the influenza B genome. Most whole-genome studies on influenza B viruses only described reassortment patterns of gene segments between lineages (or at the lineage level) [7, 13–18] and have not described reassortment patterns at the clade and sub-clade levels. The evidence for segment reassortment within a lineage or clade in contributing to worldwide disease certainly warrants a more detailed and systematic approach of phylogenetic classification of all gene segments [10]. Hence, in this study, we aimed to examine the reassortment patterns at the lineage, clade and sub-clade levels by performing phylogenetic analysis on the whole genome of influenza B/Phuket/3073/2013-like viruses and unique reassortants detected in Malaysia.

## Materials and Methods

### Ethics statement

This study was approved by the University of Malaya Medical Centre (UMMC) Medical Ethics Committee (MEC890.1). Standard, multilingual consent forms were used and written consent was obtained from all prospectively-recruited study participants. Written consent was also obtained from the next of kin, caretakers, or guardians on behalf of the minors/children enrolled in this study.

### Sample collection and detection of influenza B viruses

A total of 3,935 nasopharyngeal swab samples were prospectively collected from children and adult outpatients at the Primary Care Clinic of UMMC in Kuala Lumpur, Malaysia between 2012 and 2014 [10]. All patients experienced symptoms of acute URTI. These were tested for influenza B with the xTAG Respiratory Virus Panel (RVP) FAST multiplex RT-PCR assay (Abbott Molecular, Toronto, Canada) used with the Universal Tag sorting system on a Luminex 200 IS platform (Luminex Corp., Austin, Texas, USA), according to the manufacturer’s protocol [19]. In addition, 59 influenza B isolates from children (1 month to 15 years old) who were admitted to the hospital between January 2009 and June 2015 were also included in this study. They were obtained from the stocked cultures of the diagnostic virology laboratory of UMMC. These influenza B viruses had been cultured from nasopharyngeal aspirates in Madin-Darby canine kidney (ATCC number CCL-34) cells and confirmed by immunofluorescence assay with the Light Diagnostic Respiratory Panel 1 Viral Screening & Identification Kit (Millipore, Billerica, USA).

### Whole-genome sequencing of influenza B viruses

Using viral RNA extracted by NucliSENS easyMAG automated nucleic acid extraction system (bioMérieux, Marcy I’Etoile, France) [20] as template, all gene segments (PB2, PB1, PA, HA, NP, NA, MP and NS) of selected samples with unique HA-NA reassortment properties were amplified by two-step reverse transcription polymerase chain reaction (RT-PCR) using reagents and cycling conditions [21] listed in S1 Table. Sequences of universal primer and primer sets for all eight gene segments are listed in S2 Table. Sequencing of PCR products was performed in an ABI PRISM 3730XL Genetic Analyzer using the BigDye Terminator v3.1 cycle sequencing kit chemistry (Applied Biosystems, California, USA). Lastly, sequence reads were assembled into a contig and manually edited using BioEdit 7.2 to produce a final sequence of full-length protein-coding region of each gene segment. All sequences generated in this study are available in GenBank under accession numbers KX269900-KX270009.

### Phylogenetic analysis

In order to investigate the reassortment pattern among the selected Malaysian influenza B reassortants, phylogenetic analysis was conducted on all eight gene segments. All Malaysian sequences were first aligned with WHO recommended candidate vaccine and reference sequences, as well as selected strains from other countries retrieved from the NCBI Influenza Virus Resource [22] and the Global Initiative on Sharing all Influenza Data (GISAID) database [23] (accessed on January 14, 2016) (Table 1) using a web-based multiple sequence alignment program MAFFT [24]. Phylogenetic tree reconstructions of all genes were performed using maximum likelihood (ML) method heuristically inferred using subtree pruning and regrafting and nearest neighbor interchange algorithms with general time-reversible (GTR) nucleotide substitution model, a proportion of invariant sites (+I) and four categories of gamma rate heterogeneity (+Γ_4_), implemented in PAUP version 4.0 [25]. Robustness of the branching orders was evaluated by bootstrap analysis.

**Table 1:** Accession numbers of influenza B sequences in NCBI Influenza Virus Resource and GISAID used for the phylogenetic reconstruction of PB1, PB2, PA, HA, NP, NA, MP and NS genes.

Properties	Strains	Collection Date/Year	Accession Number for gene:							
			PB1	PB2	PA	HA	NP	NA	MP	NS
Reference	B/Lee/40	1940	DQ792895	DQ792894	DQ792896	DQ792897	DQ792898	DQ792899	DQ792900	DQ792901
Reference	B/Yamagata/16/88	1988	CY018771	CY018772	CY018770	CY018765	CY018768	CY018767	CY018766	CY018769
Reference	B/Victoria/02/1987	1987	CY018763	CY018764	CY018762	CY018757	CY018760	CY018759	CY018758	CY018761
WHO Vaccine	B/Brisbane/3/2007	2007-03-09	CY155904	CY155905	CY155903	CY155898	CY155901	CY155900	CY155899	CY155902
WHO Vaccine	B/Florida/4/2006	2006	CY033882	CY033883	CY033881	CY073895	CY033879	CY033878	CY033877	CY033880
WHO Vaccine	B/Massachusetts/02/2012	2012-03-13	EPI439263	EPI439262	EPI439261	EPI376346	EPI439258	EPI376345	EPI439260	EPI439259
WHO Vaccine	B/Phuket/3073/2013	2013-11-21	EPI544262	EPI544261	EPI547694	EPI544264	EPI544260	EPI544263	EPI592901	EPI547693
WHO Vaccine	B/Brisbane/09/2014	2014-03-24	EPI544257	EPI544256	EPI544255	EPI544259	EPI544253	EPI544258	EPI544397	EPI544254
WHO Vaccine	B/Utah/09/2014	2014-05-29	EPI544271	EPI544270	EPI544269	EPI544273	EPI544268	EPI544272	EPI544396	EPI544395
WHO Vaccine	B/Wisconsin/01/2010	2010-02-20	CY115189	CY115190	CY115188	CY115183	CY115186	CY115185	CY115184	CY115187
WHO Vaccine	B/Hubei-Wujiagang/158/2009	2009	CY115389	CY115390	CY115388	CY115383	CY115386	CY115385	CY115384	CY115387
WHO Vaccine	B/Texas/06/2011	2011-02-16	EPI354063	EPI331184	EPI331183	EPI331186	EPI354062	EPI331185	EPI331182	EPI331181
WHO Reference	B/Stockholm/12/2011	2011-02-28	-	-	-	EPI357403	-	EPI357402	-	EPI357401
WHO Vaccine	B/Bangladesh/3333/2007	2007	CY115261	CY115262	CY115260	CY115255	CY115258	CY115257	CY115256	CY115259
WHO Vaccine	B/Brisbane/33/2008	2008-07-13	EPI370452	EPI370453	EPI370451	EPI163726	EPI370450	EPI186302	EPI370449	EPI370448
WHO Vaccine	B/Brisbane/60/2008	2008-08-04	CY115157	CY115158	CY115156	CY115151	CY115154	CY115153	CY115152	CY115155
WHO Vaccine	B/Nevada/03/2011	2011-02-02	EPI354045	EPI354044	EPI354043	EPI331151	EPI354041	EPI331150	EPI354042	EPI331149
WHO Vaccine	B/Texas/02/2013	2013-01-09	EPI443688	EPI443687	EPI443686	EPI443690	EPI447191	EPI443689	EPI447193	EPI447192
WHO Vaccine	B/Bangladesh/5945/2009	2009-09-30	CY115365	CY115366	CY115364	CY115359	CY115362	CY115361	CY115360	CY115363
WHO Vaccine	B/Hong Kong/259/2010	2010	CY115197	CY115198	CY115196	CY115191	CY115194	CY115193	CY115192	CY115195
WHO Reference	B/Hong Kong/514/2009	2009-10-11	-	-	-	EPI243627	-	EPI243626	-	-
Reference	B/Bahrain/18/2013	2013-01-14	EPI491090	EPI491089	EPI491088	EPI491092	EPI491085	EPI491091	EPI491087	EPI491086
Reference	B/Thailand/CU-B6096/2012	2012-03-13	JX513099	JX513100	JX513101	JX513102	JX513103	JX513104	JX513105	JX513106
Reference	B/Thailand/CU-B6240/2012	2012-05-23	EPI635425	EPI635441	EPI635409	EPI635313	EPI635377	EPI635355	EPI635336	EPI635393
Reference	B/Nicaragua/AGB2-26/2012	2012-08-20	EPI529042	EPI528983	EPI529041	EPI529036	EPI529039	EPI529038	EPI529037	EPI529040
Reference	B/New York/1331/2012	2012-02-28	CY176007	CY176008	CY176006	CY176001	CY176004	CY176003	CY176002	CY176005
Malaysian	B/Malaysia/U1429/2012	2012-11-09	KX269974*	KX269980*	KX269986*	KR073380	KX269992*	KR073549	KX269998*	KX270004*
Malaysian	B/Malaysia/U2214/2013	2013-03-29	KX269975*	KX269981*	KX269987*	KR073421	KX269993*	KR073590	KX269999*	KX270005*
Malaysian	B/Malaysia/U2462/2013	2013-05-31	KX269976*	KX269982*	KX269988*	KR073441	KX269994*	KR073609	KX270000*	KX270006*
Malaysian	B/Malaysia/U2555/2013	2013-06-26	KX269977*	KX269983*	KX269989*	KR073446	KX269995*	KR073614	KX270001*	KX270007*
Malaysian	B/Malaysia/U3224/2013	2013-12-13	KX269978*	KX269984*	KX269990*	KR073450	KX269996*	KR073617	KX270002*	KX270008*
Malaysian	B/Malaysia/U3901/2014	2014-05-16	KX269979*	KX269985*	KX269991*	KR073493	KX269997*	KR073659	KX270003*	KX270009*

‘-’: Sequences not available;

‘*’: Sequences that were obtained in this study.

## Results and Discussion

There were 164/3,935 (children and adult outpatients) and 37/59 (hospitalized children) nasopharyngeal samples positive for HA and NA genes of influenza B viruses (S3 Table). Phylogenetic analysis of both genes identified similar circulation of Yamagata- and Victoria-lineage as well as Yam-2, Yam-3 and Vic-1 viruses between children and adult outpatients, and hospitalized children (S1 and S2 Figs, S3 Table). Furthermore, the analysis showed 36 B/Phuket/3073/2013-like viruses (28 in children and adult outpatients, 8 in hospitalized children) with intra-clade reassortment properties (HA-B/Wisconsin/01/2010-like/NA-B/Stockholm/12/2012-like) within Yam-3, one unique inter-clade reassortant (HA-Yam-2/NA-Yam-3) and one unique intra-clade reassortant within Vic-1 (HA-Vic-1B/NA-Vic-1A) (S1 and S2 Figs, S3 Table). To investigate the reassortment pattern of all eight gene segments of these reassortants from the lineage to the sub-clade level, four earliest detected B/Phuket/3073/2013-like intra-clade reassortants and the two unique reassortants sampled between 2012 and 2014 were chosen for further whole-genome analysis.

**Fig 1:**
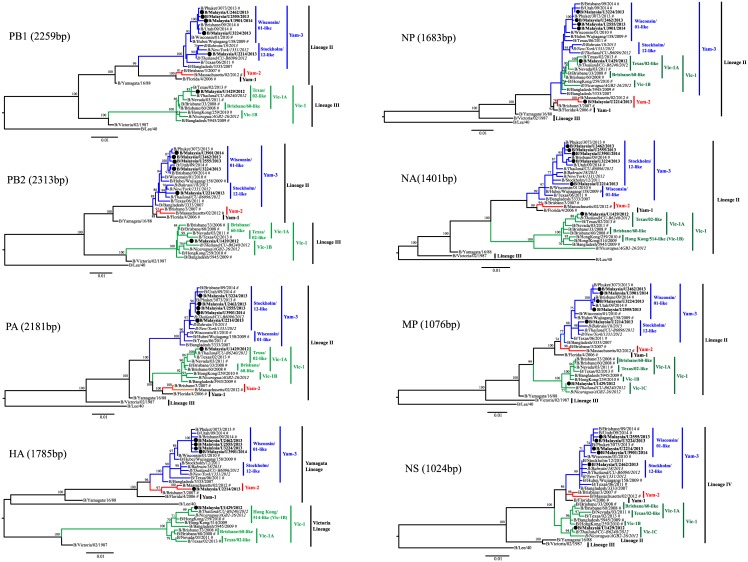
Phylogenetic comparsion based on nucleotdie sequences of PB1, PB2, PA, HA, NP, NA, MP and NS genes with that of WHO candidate vaccine (marked with #) and reference strains, Malaysian influenza B/Phuket/3073/2013-like viruses and unique reassortants (marked in black circles), and strains from different countries (in italic). Phylogenetic trees were reconstructed by maximum likelihood (ML) method with 1,000 bootstrap replicates using PAUP 4.0. Bootstrap values ≥60 are shown. Scale bar represents a genetic distance of 0.01 substitutions/site.

The phylogenetic trees reconstructed by the ML method for PB1, PB2, PA, HA, NP, NA, MP and NS genes are illustrated in Fig 1 and S3 Fig, which consist of selected Malaysian reassortants, WHO candidate vaccine and reference strains, as well as selected strains from other countries that were closely related to the selected Malaysian reassortants (≥99% sequence identities on all genes based on NCBI BLAST [26]). Consistent with other reports [7, 13, 15], the lineages for other genes (except for NS gene) were first assigned in the same manner as the HA gene, with lineages that resembled B/Yamagata/16/88-like viruses (Yam-like) designated as Lineage II, the lineages that resembled B/Victoria/2/87-like viruses (Vic-like) designated as Lineage III. The lineage that did not resemble any of the Vic- or Yam-like viruses was designated as Lineage IV (as for the case of NS gene) (Fig 1). Under such assignment, it was apparent that the tree topology for PB1, PB2, and HA genes appeared very similar, such that Yam-like and Vic-like viruses were clustered in Lineage II and III, respectively. On the other hand, the phylogenetic trees of PA, NP, NA and MP genes showed that all viruses were clustered in Lineage II (Yam-like). Furthermore, phylogenetic analysis of the NS gene showed that all viruses clustered together to form a distinct Lineage IV. The incongruent phylogenetic patterns observed at the lineage level corroborated with previous reports [7, 13, 15, 16], which suggest that the phylogenies of eight gene segments could be divided into three distinct evolutionary profiles: (i) PB1, PB2 and HA genes derived from either Lineage II or III; (ii) PA, NP, NA and MP genes derived from Lineage II; and (iii) NS genes derived from Lineage IV. Firstly, the consistent grouping of Yam-like or Vic-like viruses in Lineage II or III, respectively, in the tree topology of PB1, PB2 and HA genes could be attributed to possible genetic linkage between these three gene segments, which has been described [8]. They suggested that this pattern of co-assortment is due to the action of selection and would possibly have an effect on the whole-genome fitness of influenza B viruses [8], but the origin of this strong genetic linkage between these segments remains unclear. Secondly, for PA, NP, NA and MP gene segments, their highly similar evolutionary patterns also suggest possible functional association among the proteins [13–15], though such association remains to be investigated. Lastly, a separate lineage (Lineage IV) exhibited in the NS tree suggests that all Yam-like and Vic-like viruses possessed a NS gene segment with an unknown origin and reassortment event [7]. Taken together, the distinct evolutionary profiles shared between certain gene segments indicate that some of these genes would likely reassort together between lineages. However, whether the disappearance of a lineage in a gene segment (particularly the absence of Lineage III in PA, NP, NA and MP; Lineage II and III in NS) from the population is permanent or transient requires a large-scale analysis of genetic data.

**Fig 2:**
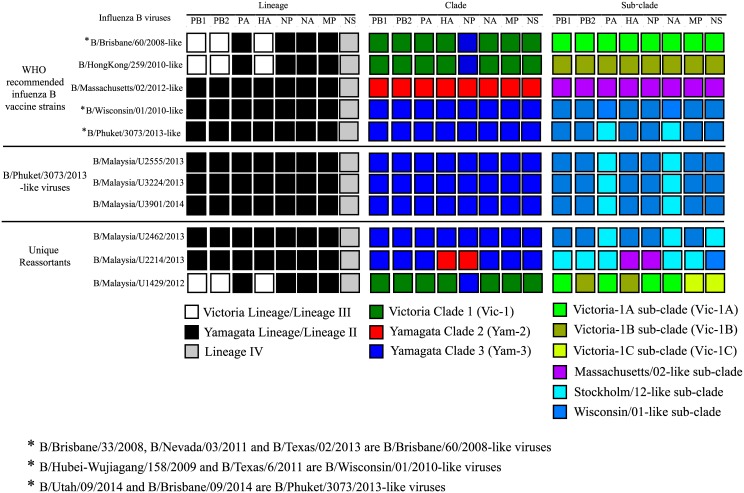
Reassortment properties of the Malaysian B/Phuket/3073/2013-like viruses and unique reassortants at the lineage, clade and sub-clade level.

The pattern of genetic reassortment became slightly less complex when influenza B viruses were phylogenetically classified into three major clades following the WHO genetic groupings [11, 12] for all gene segments: Victoria Clade 1 (Vic-1, represented by B/Brisbane/60/2008-like viruses), Yamagata Clade 1 (Yam-1, represented by B/Florida/4/2006-like viruses), Yamagata Clade 2 (Yam-2, represented by B/Brisbane/3/2007-like viruses) and Yamagata Clade 3 (Yam-3, represented by B/Bangladesh/3333/2007) (Fig 1). The tree topology showing three distinct clusters represented by major clades of Vic-1, Yam-2 and Yam-3 was similar in most of the gene segments except for the NP gene in which all Vic-1-like viruses appeared to reside within Yam-3. The sharing of a common ancestor between Vic-1-like viruses and B/Wisconsin/01/2010-like viruses (Yam-3-like viruses) in the NP gene was not observed for PA, NA and MP genes, even though previous observation at the lineage level showed that all four genes of Vic-like viruses were derived from Lineage II (Yam-like). This suggests that all Vic-1-like viruses selected in this study had an intra-lineage (or inter-clade) reassortment event that involved only the NP gene. To our knowledge, this unique reassortment property, observed in one of our Malaysian Vic-1-like virus (B/Malaysia/U1429/2012) as well as other B/Brisbane/60/2008-like viruses (Figs 1 and 2) have not been described in any reports of the whole-genome analysis of influenza B viruses.

In addition, one of our unique inter-clade reassortants (B/Malaysia/U2214/2013) which possessed HA-Yam-2/NA-Yam-3 had a genomic pattern of HA and NP that derived from Yam-2 while the rest of the gene segments were derived from Yam-3 (Figs 1 and 2). It was initially expected that PB1, PB2 and HA would derive from the same clade of Yam-2, given that a previous report suggested these three gene segments would tend to co-assort during a reassortment event [8]. As such unique reassortment pattern from B/Malaysia/U2214/2013 virus was not observed in the majority of other ‘pure’ Yam-3-like or Yam-2-like viruses (Fig 1 and S3 Fig), this phenomenon could possibly be attributed to a random intra-lineage (or inter-clade) reassortment event within the Yamagata lineage [18, 27].

Reassortment of gene segments within the major circulating clades was further investigated. Using the HA phylogeny as a reference for phylogenetic classification of other gene segments, two monophyletic clusters observed within Yam-3 were first given a designation of Wisconsin/01-like and Stockholm/12-like sub-clades [10], representing B/Wisconsin/01/2010-like and B/Stockholm/12/2011-like viruses, respectively (Fig 1). For Vic-1, two monophyletic clusters were also observed within this clade, provisionally named as Victoria-1A sub-clade (Vic-1A: represented by B/Brisbane/60/2008-like or B/Texas/02/2013-like viruses) and Victoria-1B sub-clade (Vic-1B: represented by B/Hong Kong/514/2008-like or B/Hong Kong/259/2010-like viruses) (Fig 1). Phylogenetic classification and designation of these clusters have not been widely reported as most studies and WHO interim reports on influenza vaccines did not classify influenza B viruses beyond the three major circulating clades (Yam-2, Yam-3 and Vic-1) [11–13, 28, 29]. By adapting the only influenza virus clade definition criteria developed and reported by the WHO/OIE/FAO H5N1 Evolution Working Group (WHO/OIE/FAO, 2012), phylogenetic analyses and nucleotide sequence divergence calculations of all gene segments showed that these four clusters shared a common ancestral node, had a monophyletic grouping with a bootstrap value of ≥60 and maintained an average within- and between-clade pairwise nucleotide distance of ≤1.5% and ≥1.5%, respectively. However, in the MP and NS phylogenetic trees, a unique and previously unrecognized monophyletic cluster was observed, which was provisionally assigned as Victoria-1C sub-clade (Vic-1C). Viruses within this sub-clade shared a common ancestral node with a bootstrap value of ≥70, and had an average percentage of pairwise nucleotide distances between clades (comparing with Vic-1A and Vic-1B) of 1.16 ± 0.3% and 1.46 ± 0.3%, respectively, similar to the cutoff of 1.5% set in the criteria mentioned [30]. For Yam-2, only one cluster was observed for all gene segments, namely Massachusetts/02-like sub-clade (represented by B/Massachusetts/02/2012-like viruses) (Fig 1).

Based on phylogenetic analysis of only HA and NA genes, we initially deduced that the four Malaysian viruses were B/Phuket/3073/2013-like viruses within Yam-3, due to observation of intra-clade reassortment between HA and NA [10]. However, to our surprise, when all gene segments were phylogenetically analyzed and classified at the sub-clade level, it was observed that only three Malaysian B/Phuket/3073/2013-like viruses (B/Malaysia/U2555/2013, B/Malaysia/U3224/2013 and B/Malaysia/U3901/2014) and other B/Phuket/3073/2013-like candidate vaccine strains (B/Brisbane/09/2014 and B/Utah/08/2014) were classified as intra-clade reassortants within Yam-3 (Figs 1 and 2). They possessed PB1, PB2, HA, NP, MP and NS genes that were grouped under the Wisconsin/01-like sub-clade while their PA and NA genes were grouped under the Stockholm/12-like sub-clade. As shown earlier, the evolutionary patterns of PA, NP, NA and MP genes of Vic-like and Yam-like viruses at the lineage level are similar in which they were grouped under Lineage II, suggesting possible co-assortment of these genes. However, evolutionary patterns at the sub-clade level further shows that PA and NA genes have a higher tendency to co-assort during reassortment of gene segments. Whether such co-assortment is due to a “functional linkage” between both gene segments in influenza B genome or due to a random reassortment event that increases the fitness of the virus and results in the global emergence of B/Phuket/3073/2013-like viruses remain to be elucidated.

While the other presumably B/Phuket/3073/2013-like virus (B/Malaysia/U2462/2012) had seven gene segments that were phylogenetically identical to B/Phuket/3073/2013-like viruses, the NS gene was derived from B/Stockholm/12/2011-like virus instead of B/Wisconsin/01/2010-like virus. Hence it was re-classified as a unique reassortant instead of a B/Phuket/3073/2013-like virus (Figs 1 and 2). No other B/Phuket/3073/2013-like viruses were found to share similar reassortment pattern with B/Malaysia/U2462/2012 (S3 Fig). For the other unique reassortants found based on phylogenetic analysis of HA and NA genes, the inter-clade reassortant (B/Malaysia/U2214/2013 which had HA-Yam-2-like/NA-Yam-3-like) had a NS gene that derived from B/Wisconsin/01/2010-like virus instead of B/Stockholm/12/2011-like virus. In addition, the Vic-like inter-lineage reassortant (B/Malaysia/U1429/2012) also showed inter- and intra-clade reassortment properties (PB2 and HA derived from Vic-1B; PB1, PA, NP, and NA from Vic-1A; MP and NS from Vic-1C). This pattern was also observed in only one Asian virus, B/Thailand/CU-B6240/2012, which shared identical reassortment properties with B/Malaysia/U1429/2012 (Fig 1 and S3 Fig). With that, the phylogeny of each gene segments observed at the sub-clade level in these unique reassortants suggests that a random reassortment event within a clade may occur and these reassortants are probably circulating at a very low level [18, 27]. Of note, the overall genetic makeup of these unique reassortants were distinct from the WHO recommended Yamagata-like (B/Wisconsin/01/2010-like in 2013, B/Massachusetts/02/2012 in 2014, B/Phuket/3073/2013 in 2015 and 2016) and Victoria-like (B/Brisbane/60/2008-like from 2012 to 2016 and B/Hong Kong/259/2010-like from 2014 to 2016) vaccine strains for the Southern Hemisphere (Fig 2). As information regarding the vaccine uptake of patients infected by these unique reassortants were not recorded, whether such reassortants caused more severe disease than the predominant circulating strains with enhanced viral fitness or whether they were able to be recognized effectively by antibodies generated by the recommended vaccine strains remains in question. Hence, influenza surveillance coupled with phylogenetic analyses should be intensified in order to detect more low-level circulating unique reassortants that may contribute to future outbreaks.

In conclusion, this present study highlights the strategy used for the phylogenetic classification of all gene segments of influenza B viruses, which classifies all genes at the lineage, clade and sub-clade level. This strategy for lineage classification was adapted from previously reported methods [7, 13, 15, 16], while the strategy for clade and sub-clade classification was adapted from the WHO interim reports [11, 12] and criteria used for H5N1 classification [30], respectively. Adaptation of this approach in future studies with a larger dataset of whole-genome sequences may perhaps allow a better understanding of the complexity of segment reassortments in influenza B evolution which may contribute to future vaccine design, and also for better monitoring of unique reassortants in surveillance studies.

## Supporting Information

S1 Fig Phylogenetic anlaysis of the HA gene of influenza B viruses in Malaysia using the maximum likelihood (ML) method with 1,000 bootstrap replicates using PAUP 4.0 with complete taxa identity. Bootstrap values ≥60 are shown. Malaysian B/Phuket/3073/2013-like viruses and unique reassortants are highlighted in bold and purple. Scale bar represents a genetic distance of 0.01 substitutions/site.
(PDF)

S2 Fig Phylogenetic anlaysis of the NA gene of influenza B viruses in Malaysia using the maximum likelihood (ML) method with 1,000 bootstrap replicates using PAUP 4.0 with complete taxa identity. Bootstrap values ≥60 are shown. Malaysian B/Phuket/3073/2013-like viruses and unique reassortants are highlighted in bold and purple. Scale bar represents a genetic distance of 0.01 substitutions/site.
(PDF)

S3 Fig Phylogenetic analyses of the HA, NA, PB1, PB2, PA, NP, MP and NS genes using the maximum likelihood (ML) method with 1,000 bootstrap replicates using PAUP 4.0 to detect for global viruses that share similar reassortment pattern with the Malaysian influenza B unique reassortants. Analyses began by retrieving complete genomes of 6,832 influenza B isolates from GISAID database. With this set of data, phylogenetic analysis on the HA gene was performed and a total of 51 strains were found to be closely related (by forming a cluster and have ≥99% sequence identities in BLAST) with our reassortants. These 51 strains were then used as references for all gene segments. Bootstrap values ≥60 are shown. Malaysian B/Phuket/3073/2013-like viruses and unique reassortants are highlighted in bold. Scale bar represents a genetic distance of 0.01 substitutions/site.
(PDF)

S1 Table Reagents and thermocycling conditions used for two-step reverse transcription polymerase chain reaction (RT-PCR) for amplification of influenza B PB1, PB2, PA, HA, NP, NA, MP and NS genes. (DOCX)

S2 Table Primer sets used for the PCR amplification of PB2, PB1, PA, HA, NP, NA, MP and NS genes of influenza B/Phuket/3073/2013-like viruses and unique reassortants detected in Malaysia. (DOCX)

S3 Table HA and NA genes of influenza B virus clinical isolates sequenced in previous and in this study. (DOCX)
