# Supplementary material for: Whole-Genome Phylogenetic Analysis of Influenza B/Phuket/3073/2013-Like Viruses and Unique Reassortants Detected in Malaysia between 2012 and 2014
Source: PLoS One. 2017 Jan 27;12(1):e0170610. doi: 10.1371/journal.pone.0170610 (PMC5271328; doi:10.1371/journal.pone.0170610)
Supplement: S1 Fig — Bootstrap values ≥60 are shown. Malaysian B/Phuket/3073/2013-like viruses and unique reassortants are highlighted in bold and purple. Scale bar represents a genetic distance of 0.01 substitutions/site. (PDF) [file pone.0170610.s001.pdf]

S1 Fig

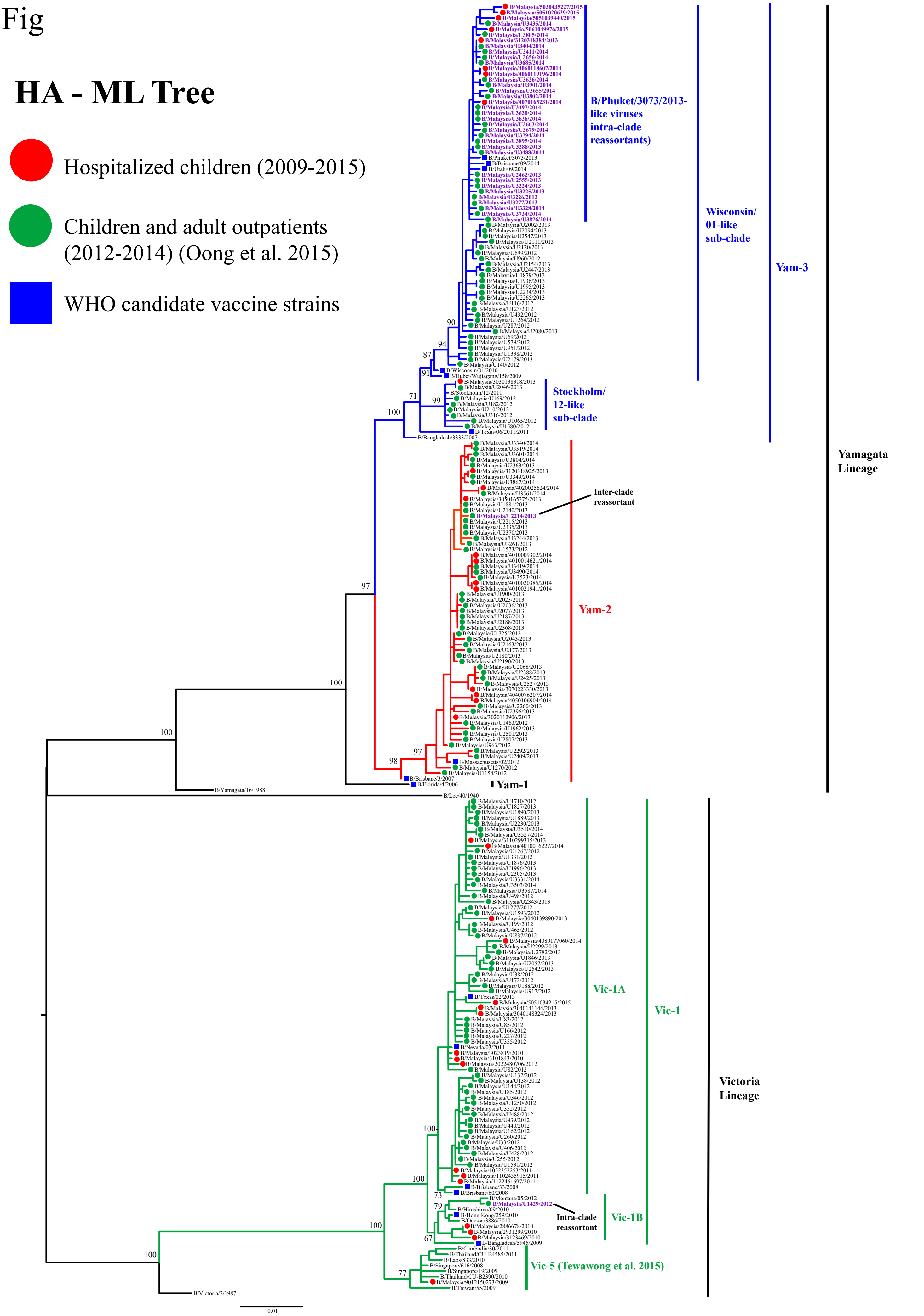
HA - ML Tree
Hospitalized children (2009-2015)
Children and adult outpatients (2012-2014) (Oong et al. 2015)
WHO candidate vaccine strains
B/Phuket/3073/2013-like viruses intra-clade reassortants)
Wisconsin/ 01-like sub-clade
Yam-3
Stockholm/ 12-like sub-clade
Yamagata Lineage
Inter-clade reassortant
Yam-2
Yam-1
B/Yamagata/16/1988
B/Lee/40/1940
Vic-1A
Vic-1
Victoria Lineage
Intra-clade reassortant
Vic-1B
Vic-5 (Tewawong et al. 2015)
B/Victoria/2/1987
0.01
100
97
100
100
71
99
91
87
94
90
98
97
100
100
73
79
67
100
100
77
