# Supplementary figures and images for: Whole-Genome Phylogenetic Analysis of Influenza B/Phuket/3073/2013-Like Viruses and Unique Reassortants Detected in Malaysia between 2012 and 2014
Source: PLoS One. 2017 Jan 27;12(1):e0170610. doi: 10.1371/journal.pone.0170610 (PMC5271328; doi:10.1371/journal.pone.0170610)

## NA - ML Tree

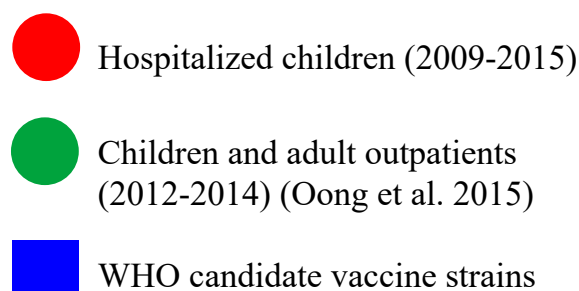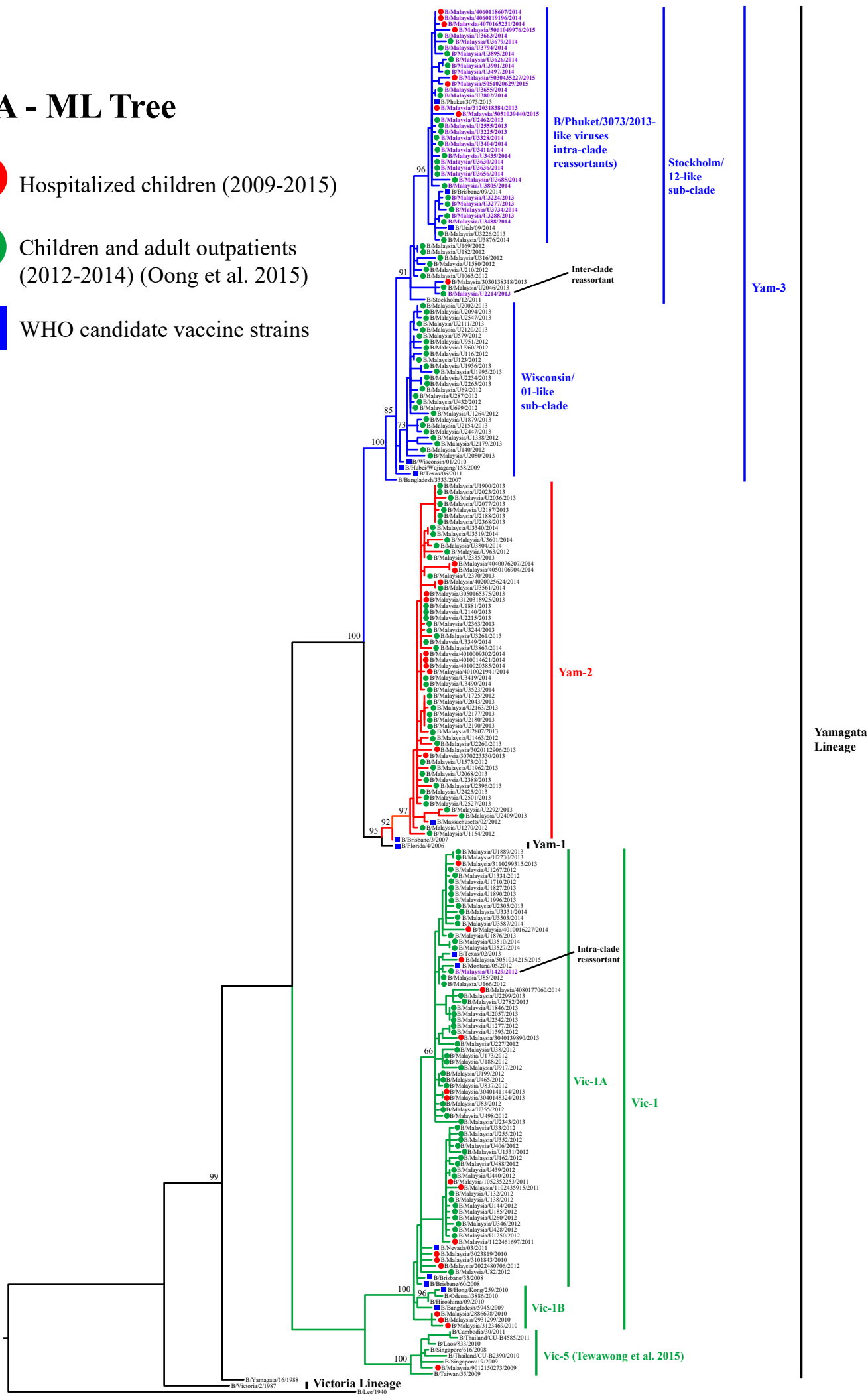

Supplement: S2 Fig — Bootstrap values ≥60 are shown. Malaysian B/Phuket/3073/2013-like viruses and unique reassortants are highlighted in bold and purple. Scale bar represents a genetic distance of 0.01 substitutions/site. (PDF) [file pone.0170610.s002.pdf]
