# Supplementary material for: Whole-Genome Phylogenetic Analysis of Influenza B/Phuket/3073/2013-Like Viruses and Unique Reassortants Detected in Malaysia between 2012 and 2014
Source: PLoS One. 2017 Jan 27;12(1):e0170610. doi: 10.1371/journal.pone.0170610 (PMC5271328; doi:10.1371/journal.pone.0170610)

S3 Fig

ML Tree - HA (1785bp)

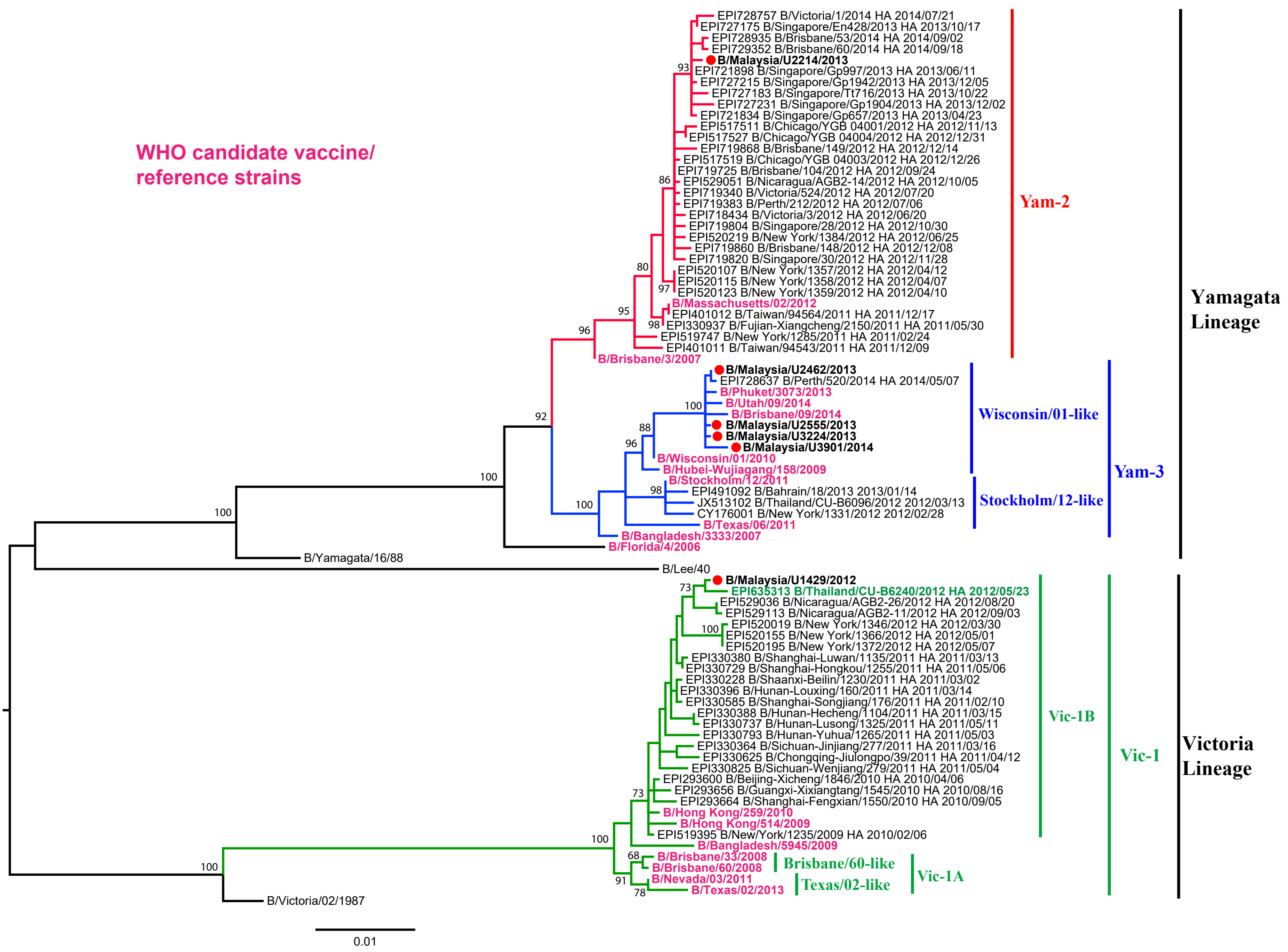

ML Tree - NA (1401bp)

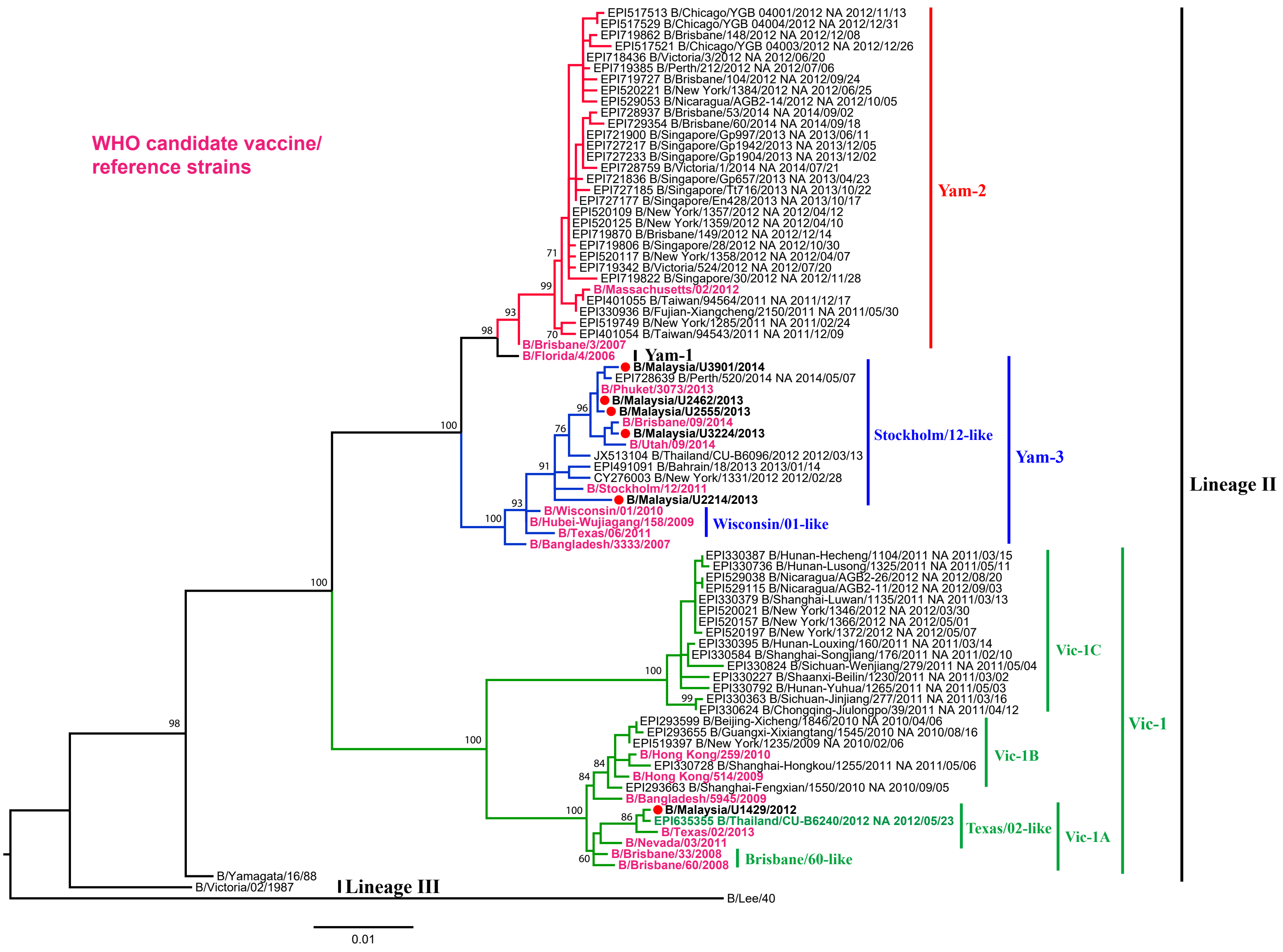

ML Tree - PB1 (2259bp)

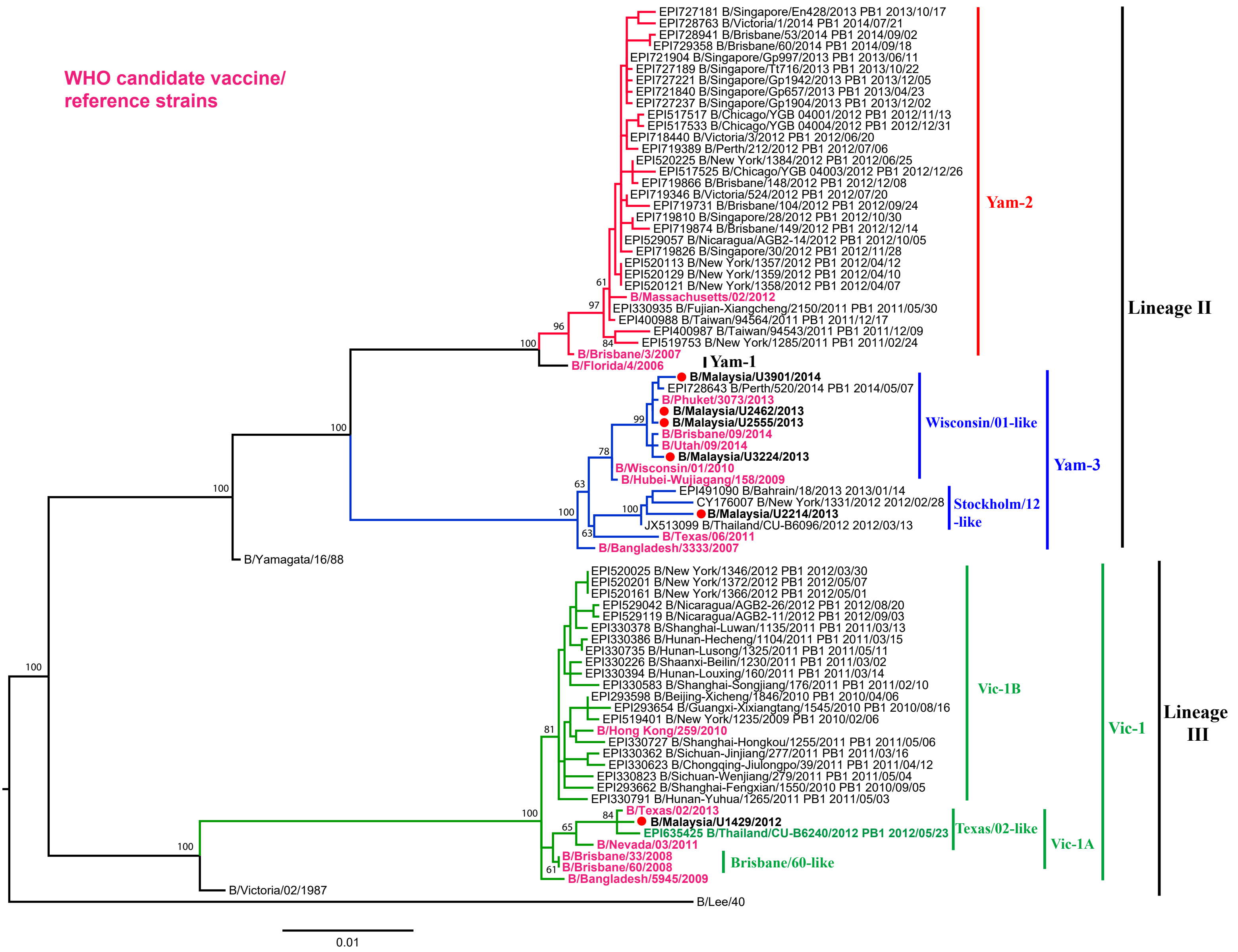

ML Tree - PB2 (2313bp)

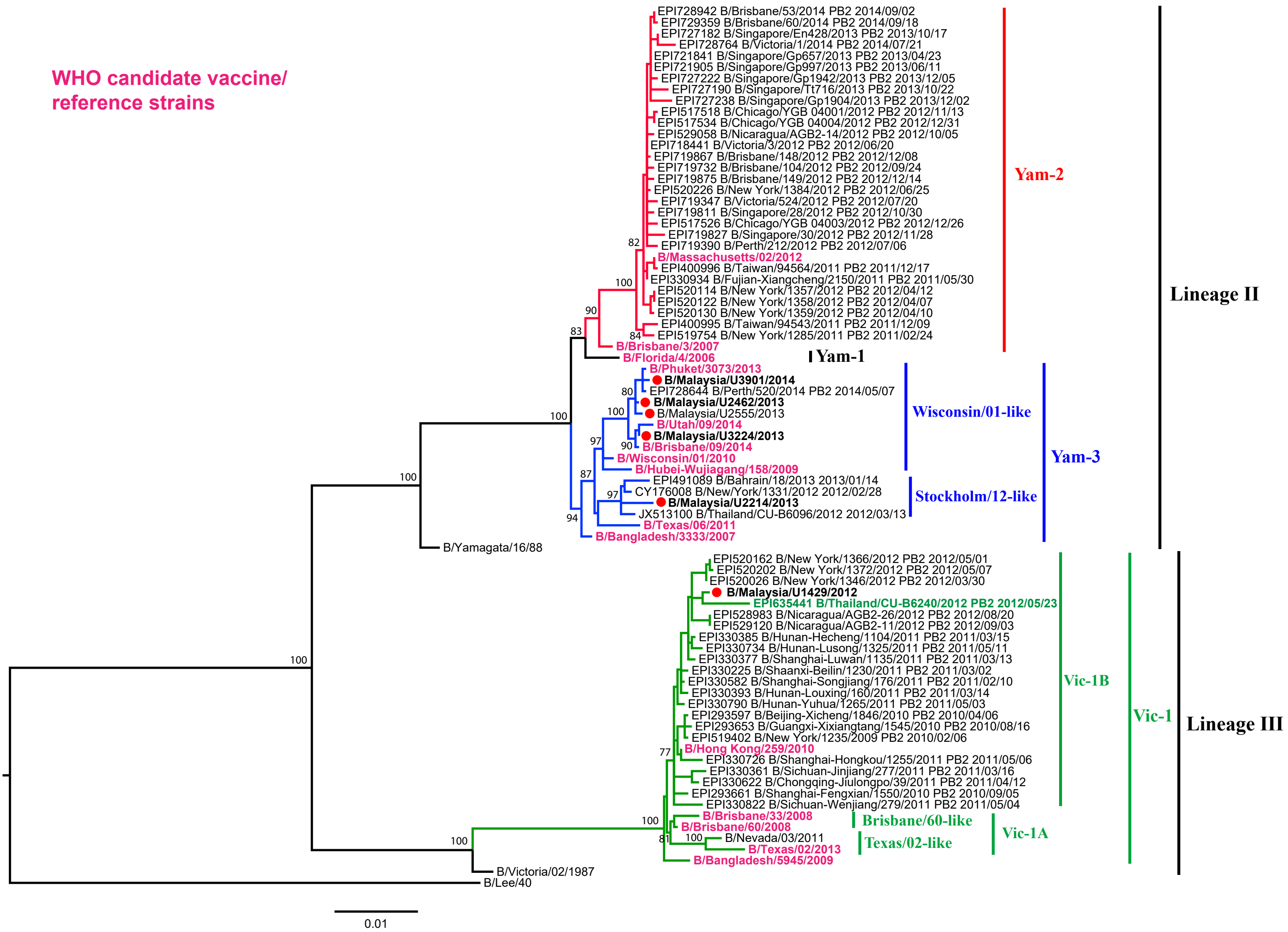

ML Tree - PA (2181bp)

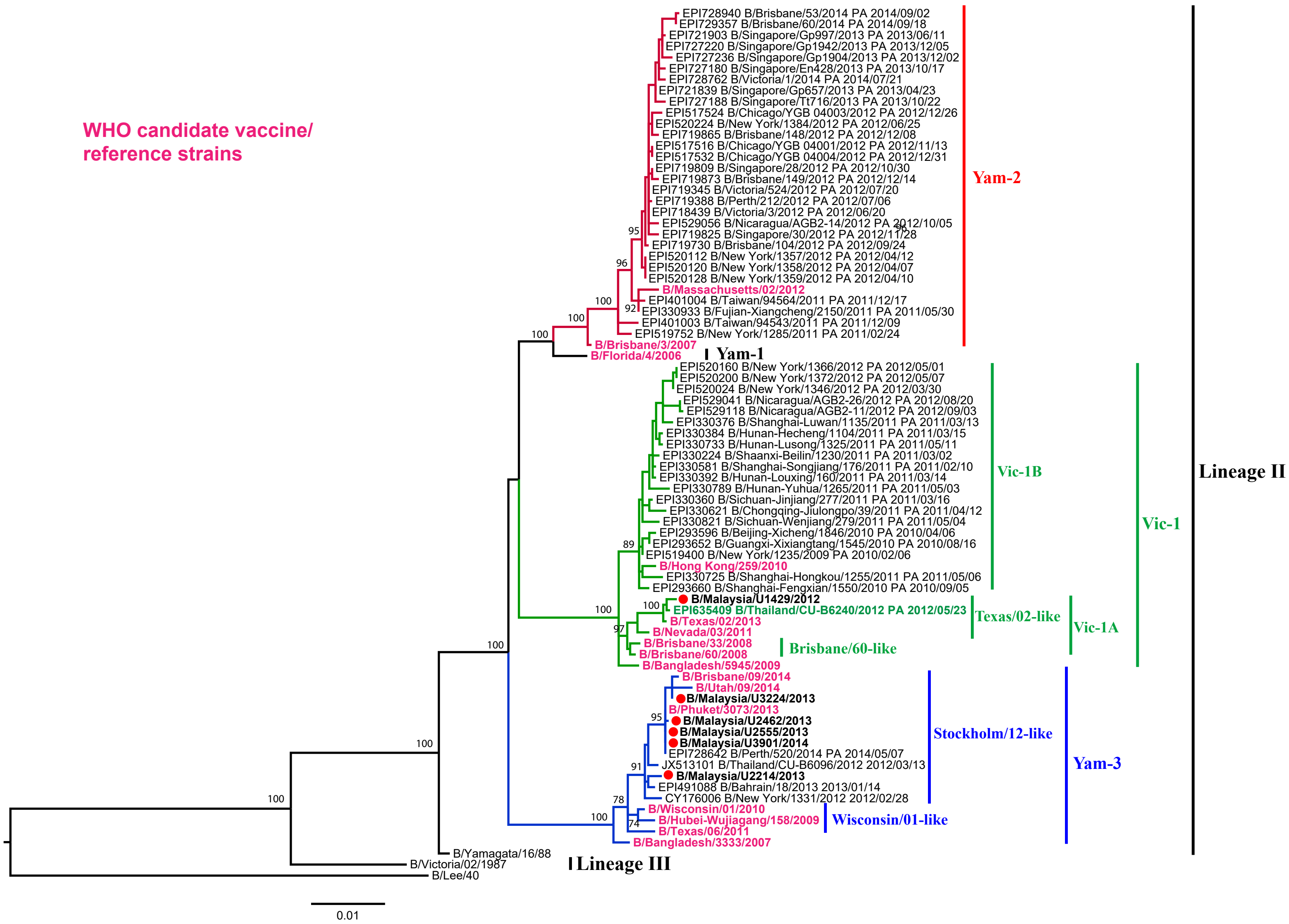

ML Tree - NP (1683bp)

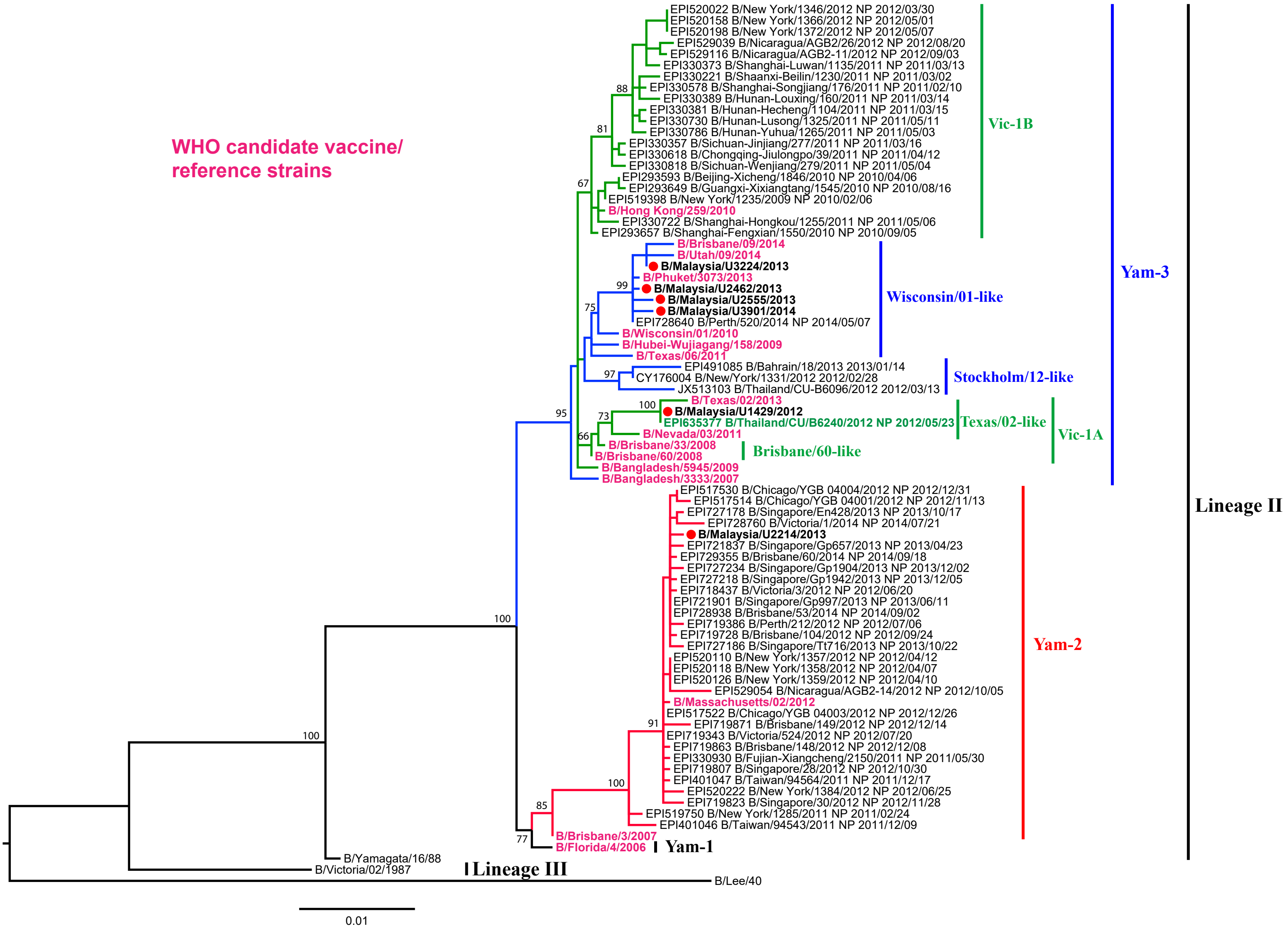

ML Tree - MP (1076bp)

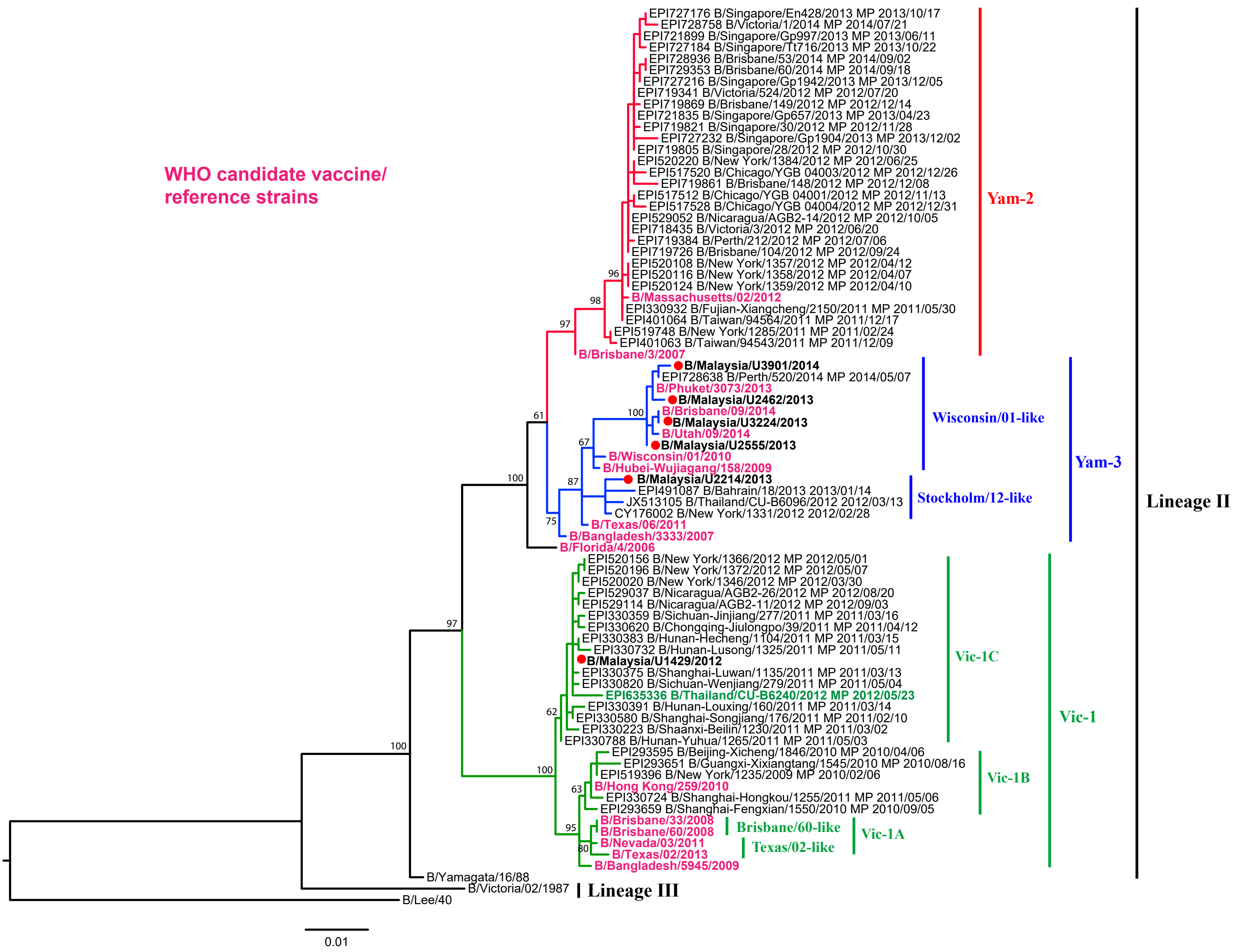

ML Tree - NS (1024bp)

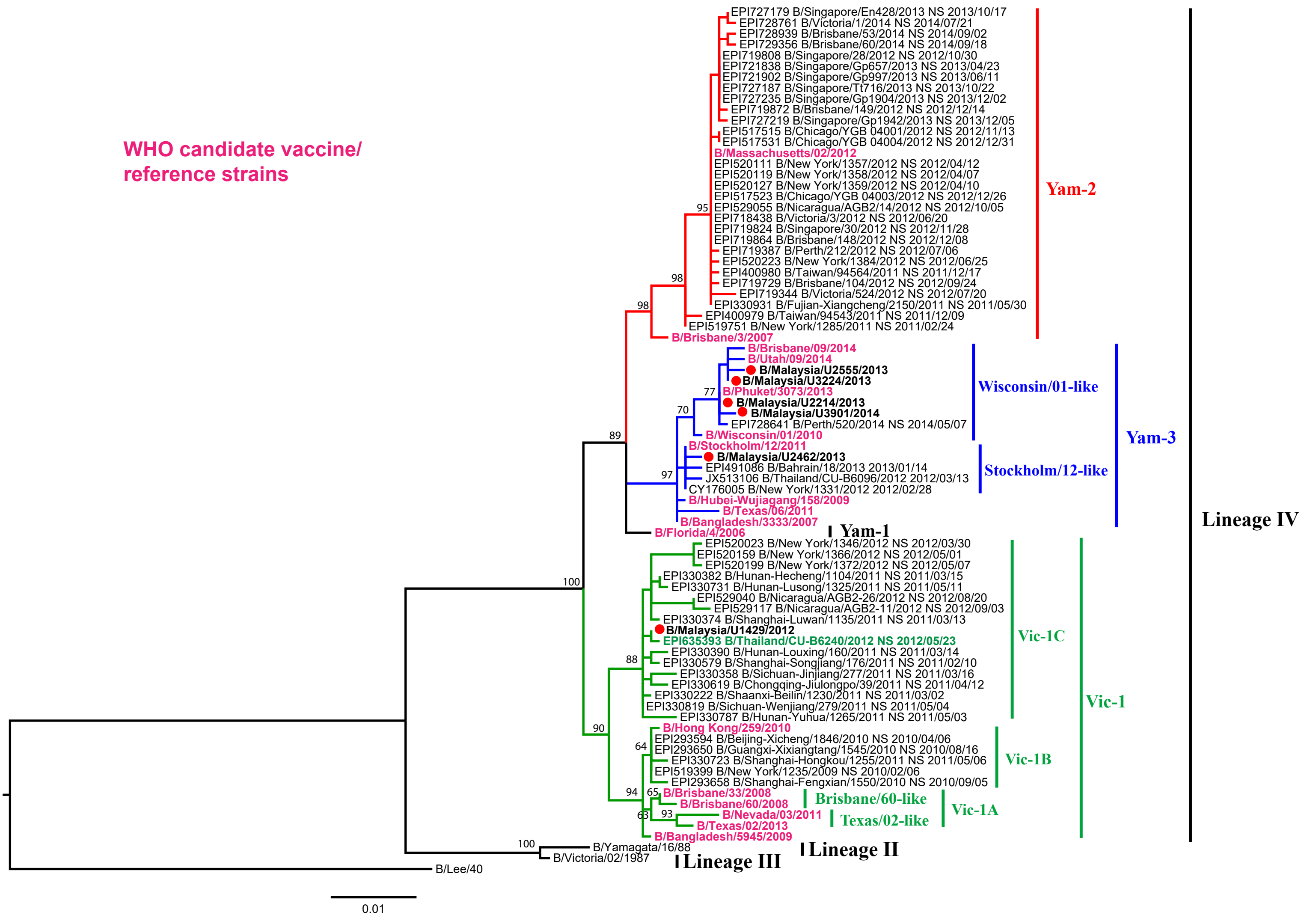

Supplement: S3 Fig — Analyses began by retrieving complete genomes of 6,832 influenza B isolates from GISAID database. With this set of data, phylogenetic analysis on the HA gene was performed and a total of 51 strains were found to be closely related (by forming a cluster and have ≥99% sequence identities in BLAST) with our reassortants. These 51 strains were then used as references for all gene segments. Bootstrap values ≥60 are shown. Malaysian B/Phuket/3073/2013-like viruses and unique reassortants are highlighted in bold. Scale bar represents a genetic distance of 0.01 substitutions/site. (PDF) [file pone.0170610.s003.pdf]
