# Supplementary material for: Whole-Genome Phylogenetic Analysis of Influenza B/Phuket/3073/2013-Like Viruses and Unique Reassortants Detected in Malaysia between 2012 and 2014
Source: PLoS One. 2017 Jan 27;12(1):e0170610. doi: 10.1371/journal.pone.0170610 (PMC5271328; doi:10.1371/journal.pone.0170610)
Supplement: S1 Table — (DOCX) [file pone.0170610.s004.docx]

**S1 Table.** **Reagents and thermocycling conditions used for two-step reverse-transcription polymerase chain reaction (RT-PCR) for amplification of influenza B PB1, PB2, PA, HA, NP, NA, MP and NS genes.**

1. Reagents and their volumes used for reverse transcription (RT) step.

| **Reagent** | **Vol. (1X), μl** |
| --- | --- |
| Universal Primer (Buni11W) | 4.5 |
| Template (RNA) | 10.0 |
| Water (Molecular Grade) | 12.6 |
| 5x First-Strand Buffer* | 8.0 |
| 0.1M DTT* | 2.0 |
| 100mM dNTP* | 0.9 |
| Superscript^TM^ III Reverse Transcriptase | 2.0 |
| **Total** | **40.0** |

* Invitrogen^TM^, Life Technologies, USA

1. Thermocycling conditions for reverse transcription (RT) step.

| **Thermocycling steps** | **Temperature** | **Cycle** | **Time (Min)** |
| --- | --- | --- | --- |
| Denaturation and  primer annealing | Mix primer and RNA.  Incubate at 65°C.  Then chilled on ice | 1 | 5 |
| Enzyme activation | 25°C | 1 | 5 |
| Extension | 50°C | 1 | 60 |
| Enzyme deactivation | 70°C | 1 | 15 |

1. Reagents and their volumes used for polymerase chain reaction (PCR) step.

| **Reagent** | **Vol. (1X), μl** |
| --- | --- |
| 10x PCR Buffer (15mM MgCl_2_)* | 5.00 |
| dNTP mix (10mM of each) | 1.00 |
| Primer F | 2.00 |
| Primer R | 2.00 |
| HotStarTaq *Plus* DNA Polymerase (250 units) | 0.25 |
| RNase-free water | 35.75 |
| RT product | 4.00 |
| **Total** | **50.0** |

* QIAGEN^®^, Germany

1. Thermocycling conditions for polymerase chain reaction (PCR) step.

| **Thermocycling steps** | **Temperature** | **Cycle** | **Time (Min)** |
| --- | --- | --- | --- |
| Initial activation | 95 | 1 | 5 |
| Denaturation | 94 | 40 | 0.5 |
| Annealing | 55 |  | 1 |
| Extension | 68 |  | 1.5 |
| Final Extension | 68 | 1 | 10 |
