# Supplementary material for: Whole-Genome Phylogenetic Analysis of Influenza B/Phuket/3073/2013-Like Viruses and Unique Reassortants Detected in Malaysia between 2012 and 2014
Source: PLoS One. 2017 Jan 27;12(1):e0170610. doi: 10.1371/journal.pone.0170610 (PMC5271328; doi:10.1371/journal.pone.0170610)
Supplement: S2 Table — (DOCX) [file pone.0170610.s005.docx]

**S2 Table.** **Primer sets used for the PCR amplification of PB2, PB1, PA, HA, NP, NA, MP and NS genes of influenza B/Phuket/3073/2013-like viruses and unique reassortants detected in Malaysia.**

| **Gene** | **Primer Name** | **Nucleotide Sequence (5' - 3')** | **PCR product (bp)** | **Total Size (bp)** |
| --- | --- | --- | --- | --- |
|  |  |  |  |  |
| All | Buni11W# | AGC AGA AGC GS | - | - |
| PB2 | PB2_P1F* | AGC AGA AGC GGA GC**G** TTT TCA AG | 1105 | 2313 |
|  | PB2_P2R* | CCG TCC CAT ATT CCA ATC TTC TG |  |  |
|  | PB2_P3F* | CAG C**Y**A TAG ACG GAG GTG ATG T | 995 |  |
|  | PB2_P4R* | TGA TA**R** GGC TCC CCA TTG CTC CTT |  |  |
|  | PB2_P5F* | GTT CCA ATG GGA TGC ATT TGA AG | 658 |  |
|  | PB2_P6R | AGT AGA AAC ACG AGC ATT TTT C |  |  |
| PB1 | PB1_P1F* | AGC AGA AGC GGA GCC TTT AAG ATG | 1043 | 2259 |
|  | PB1_P2R* | C**M**G GTG CTA TAC TAC AAA AAT CCC |  |  |
|  | PB1_P3F* | GCA TGA CAG TAA CAG GAG ACA AT | 1051 |  |
|  | PB1_P4R* | CCA TGT GCT GG**R** GTT AT**R** TCT GC |  |  |
|  | PB1_P5F* | ACA C**M**T ACA AAT GCC ACA G**R**G GAG | 677 |  |
|  | PB1_P6R | AGT AGA AAC ACG AGC **C**TT **TTT C** |  |  |
| PA | PA_P1F* | AGC AGA AGC GGT GCG TTT GAT TTG | 1135 | 2181 |
|  | PA_P2R* | ATG TTA A**W**C CAT CCC CTG TGG CCC |  |  |
|  | PA_P3F* | GGA A**R**C TTT GGA GAG ACT GTG TAA A | 822 |  |
|  | PA_P4R* | GGG GCT ATT TAC TCT GTC **Y**CC C |  |  |
|  | PA_P5F* | TTG GCT CCC TAT TTG TGA GTG G**R** | 655 |  |
|  | PA_P6R* | AGT AGA AAC ACG TGC ATT TTT G |  |  |
| HA | BHAF1U# | AGC AGA AGC AGA GCA TTT TCT AAT ATC | 1361 | 1758 |
|  | BHAR1341# | TTC GTT GTG GAG TTC ATC CAT |  |  |
|  | BHAF458# | AGA AAA GGC ACC AGG AGG ACC CTA | 1391 |  |
|  | BHA2R1# | GTA ATG GTA ACA AGC AAA CAA GCA |  |  |
| NP | NP_P1F* | AGC AGA AG**C** ACA GCA TTT TCT TG | 998 | 1683 |
|  | NP_P2R* | TCT TCA ATG TCT GCA ATC CC**Y** GG |  |  |
|  | NP_P3F* | GGC AGA **Y**AG AGG GCT ATT GAG AG | 992 |  |
|  | NP_P4R* | AGT AGA AAC AAC AGC ATT TTT TA |  |  |
| NA | BNAF1U# | AGC AGA AGC AGA GCA TCT TCT CA | 1130 | 1401 |
|  | BNAR2# | GAT GGA CAA ATC CTC CCT TGA TGC |  |  |
|  | BNAF2# | GCA CTC CTA ATT AGC CCT CAT AGA | 1182 |  |
|  | BNAR1487# | TAA GGA CAA TTG TTC AAA C |  |  |
| MP | M_P1F* | A**G**C AGA AGC ACG CAC TTT CTT AA | 835 | 1076 |
|  | M_P2R | TTG TCC ARG CCA TGA AAT GGA |  |  |
|  | M_P3F | ATG GAA GTG CTA AAG CAG AGC TC | 479 |  |
|  | M_P4R | AGT AGA AAC AAC GCA CTT TTT C |  |  |
| NS | NS_P1F* | AGC AGA AGC AGA GGA TTT GTT TAG | 751 | 1024 |
|  | NS_P2R | TCT TCT TCA TCC TCC ACT GTA |  |  |
|  | NS_P3F | GGG ACA TGA ACA ACA AAG ATG | 610 |  |
|  | NS_P4R | AGT AGT AAC AAG AGG ATT TTT A |  |  |

* Primer sequences adopted from previous study [1]. Bold and underlined letters indicate modified nucleotide sequences for better sequence coverage. # Primer sequences obtained from WHO information for molecular diagnosis of influenza virus [2].

**References**

1. Tewawong N, Suwannakarn K, Prachayangprecha S, Korkong S, Vichiwattana P, Vongpunsawad S, et al. Molecular epidemiology and phylogenetic analyses of influenza B virus in Thailand during 2010 to 2014. PLoS One. 2015;10(1):e0116302. doi:10.1371/journal.pone.0116302

2. WHO. WHO information for molecular diagnosis of influenza virus. Updated March 2014. 2014.
