# Supplementary material for: Whole-Genome Phylogenetic Analysis of Influenza B/Phuket/3073/2013-Like Viruses and Unique Reassortants Detected in Malaysia between 2012 and 2014
Source: PLoS One. 2017 Jan 27;12(1):e0170610. doi: 10.1371/journal.pone.0170610 (PMC5271328; doi:10.1371/journal.pone.0170610)
Supplement: S3 Table — (DOCX) [file pone.0170610.s006.docx]

**S3 Table.** **HA and NA genes of influenza B virus clinical isolates sequenced in previous and in this study.**

| **No** | **Name of Sample** | **Age** | **Lineage** | **Clade** | | | | **Source** |
| --- | --- | --- | --- | --- | --- | --- | --- | --- |
|  |  |  |  | **Accession No. (HA)** | **HA** | **Accession No. (NA)** | **NA** |  |
| 1 | B/Malaysia/U33/2012 | 41 | Victoria | KR073326 | Vic-1 (Vic-1A) | KR073494 | Vic-1 (Vic-1A) | [1] |
| 2 | B/Malaysia/U38/2012 | 65 | Victoria | KR073327 | Vic-1 (Vic-1A) | KR073495 | Vic-1 (Vic-1A) |  |
| 3 | B/Malaysia/U69/2012 | 13 | Yamagata | KR073328 | Yam-3 (Wisconsin/01) | KR073496 | Yam-3 (Wisconsin/01) |  |
| 4 | B/Malaysia/U82/2012 | 65 | Victoria | KR073329 | Vic-1 (Vic-1A) | KR073497 | Vic-1 (Vic-1A) |  |
| 5 | B/Malaysia/U83/2012 | 28 | Victoria | KR073330 | Vic-1 (Vic-1A) | KR073498 | Vic-1 (Vic-1A) |  |
| 6 | B/Malaysia/U85/2012 | 13 | Victoria | KR073331 | Vic-1 (Vic-1A) | KR073499 | Vic-1 (Vic-1A) |  |
| 7 | B/Malaysia/U116/2012 | 51 | Yamagata | KR073332 | Yam-3 (Wisconsin/01) | KR073500 | Yam-3 (Wisconsin/01) |  |
| 8 | B/Malaysia/U123/2012 | 51 | Yamagata | KR073333 | Yam-3 (Wisconsin/01) | KR073501 | Yam-3 (Wisconsin/01) |  |
| 9 | B/Malaysia/U132/2012 | 16 | Victoria | KR073334 | Vic-1 (Vic-1A) | KR073502 | Vic-1 (Vic-1A) |  |
| 10 | B/Malaysia/U138/2012 | 52 | Victoria | KR073335 | Vic-1 (Vic-1A) | KR073503 | Vic-1 (Vic-1A) |  |
| 11 | B/Malaysia/U140/2012 | 46 | Yamagata | KR073336 | Yam-3 (Wisconsin/01) | KR073504 | Yam-3 (Wisconsin/01) |  |
| 12 | B/Malaysia/U144/2012 | 13 | Victoria | KR073337 | Vic-1 (Vic-1A) | KR073505 | Vic-1 (Vic-1A) |  |
| 13 | B/Malaysia/U162/2012 | 26 | Victoria | KR073338 | Vic-1 (Vic-1A) | KR073507 | Vic-1 (Vic-1A) |  |
| 14 | B/Malaysia/U166/2012 | 32 | Victoria | KR073339 | Vic-1 (Vic-1A) | KR073508 | Vic-1 (Vic-1A) |  |
| 15 | B/Malaysia/U169/2012 | 45 | Yamagata | KR073340 | Yam-3 (Stockholm/12) | KR073509 | Yam-3 (Stockholm/12) |  |
| 16 | B/Malaysia/U173/2012 | 27 | Victoria | KR073341 | Vic-1 (Vic-1A) | KR073510 | Vic-1 (Vic-1A) |  |
| 17 | B/Malaysia/U182/2012 | 72 | Yamagata | KR073342 | Yam-3 (Stockholm/12) | KR073511 | Yam-3 (Stockholm/12) |  |
| 18 | B/Malaysia/U185/2012 | 34 | Victoria | KR073343 | Vic-1 (Vic-1A) | KR073512 | Vic-1 (Vic-1A) |  |
| 19 | B/Malaysia/U188/2012 | 26 | Victoria | KR073344 | Vic-1 (Vic-1A) | KR073513 | Vic-1 (Vic-1A) |  |
| 20 | B/Malaysia/U199/2012 | 35 | Victoria | KR073345 | Vic-1 (Vic-1A) | KR073514 | Vic-1 (Vic-1A) |  |
| 21 | B/Malaysia/U210/2012 | 11 | Yamagata | KR073346 | Yam-3 (Stockholm/12) | KR073515 | Yam-3 (Stockholm/12) |  |
| 22 | B/Malaysia/U227/2012 | 24 | Victoria | KR073347 | Vic-1 (Vic-1A) | KR073516 | Vic-1 (Vic-1A) |  |
| 23 | B/Malaysia/U255/2012 | 19 | Victoria | KR073348 | Vic-1 (Vic-1A) | KR073518 | Vic-1 (Vic-1A) |  |
| 24 | B/Malaysia/U260/2012 | 23 | Victoria | KR073349 | Vic-1 (Vic-1A) | KR073519 | Vic-1 (Vic-1A) |  |
| 25 | B/Malaysia/U287/2012 | 38 | Yamagata | KR073350 | Yam-3 (Wisconsin/01) | KR073520 | Yam-3 (Wisconsin/01) |  |
| 26 | B/Malaysia/U316/2012 | 49 | Yamagata | KR073351 | Yam-3 (Stockholm/12) | KR073521 | Yam-3 (Stockholm/12) |  |
| 27 | B/Malaysia/U346/2012 | 54 | Victoria | KR073352 | Vic-1 (Vic-1A) | KR073522 | Vic-1 (Vic-1A) |  |
| 28 | B/Malaysia/U352/2012 | 7 | Victoria | KR073353 | Vic-1 (Vic-1A) | KR073523 | Vic-1 (Vic-1A) |  |
| 29 | B/Malaysia/U355/2012 | 14 | Victoria | KR073354 | Vic-1 (Vic-1A) | KR073524 | Vic-1 (Vic-1A) |  |
| 30 | B/Malaysia/U406/2012 | 58 | Victoria | KR073355 | Vic-1 (Vic-1A) | KR073525 | Vic-1 (Vic-1A) |  |
| 31 | B/Malaysia/U428/2012 | 17 | Victoria | KR073356 | Vic-1 (Vic-1A) | KR073526 | Vic-1 (Vic-1A) |  |
| 32 | B/Malaysia/U432/2012 | 57 | Yamagata | KR073357 | Yam-3 (Wisconsin/01) | KR073527 | Yam-3 (Wisconsin/01) |  |
| 33 | B/Malaysia/U439/2012 | 20 | Victoria | KR073358 | Vic-1 (Vic-1A) | KR073528 | Vic-1 (Vic-1A) |  |
| 34 | B/Malaysia/U440/2012 | 20 | Victoria | KR073359 | Vic-1 (Vic-1A) | KR073529 | Vic-1 (Vic-1A) |  |
| 35 | B/Malaysia/U465/2012 | 30 | Victoria | KR073360 | Vic-1 (Vic-1A) | KR073530 | Vic-1 (Vic-1A) |  |
| 36 | B/Malaysia/U488/2012 | 37 | Victoria | KR073361 | Vic-1 (Vic-1A) | KR073531 | Vic-1 (Vic-1A) |  |
| 37 | B/Malaysia/U498/2012 | 37 | Victoria | KR073362 | Vic-1 (Vic-1A) | KR073532 | Vic-1 (Vic-1A) |  |
| 38 | B/Malaysia/U579/2012 | 68 | Yamagata | KR073363 | Yam-3 (Wisconsin/01) | KR073533 | Yam-3 (Wisconsin/01) |  |
| 39 | B/Malaysia/U699/2012 | 72 | Yamagata | KR073364 | Yam-3 (Wisconsin/01) | KR073534 | Yam-3 (Wisconsin/01) |  |
| 40 | B/Malaysia/U837/2012 | 29 | Victoria | KR073366 | Vic-1 (Vic-1A) | KR073535 | Vic-1 (Vic-1A) |  |
| 41 | B/Malaysia/U917/2012 | 63 | Victoria | KR073367 | Vic-1 (Vic-1A) | KR073536 | Vic-1 (Vic-1A) |  |
| 42 | B/Malaysia/U951/2012 | 23 | Yamagata | KR073368 | Yam-3 (Wisconsin/01) | KR073537 | Yam-3 (Wisconsin/01) |  |
| 43 | B/Malaysia/U960/2012 | 12 | Yamagata | KR073369 | Yam-3 (Wisconsin/01) | KR073538 | Yam-3 (Wisconsin/01) |  |
| 44 | B/Malaysia/U963/2012 | 46 | Yamagata | KR073370 | Yam-2 | KR073539 | Yam-2 |  |
| 45 | B/Malaysia/U1065/2012 | 13 | Yamagata | KR073371 | Yam-3 (Stockholm/12) | KR073540 | Yam-3 (Stockholm/12) |  |
| 46 | B/Malaysia/U1154/2012 | 49 | Yamagata | KR073372 | Yam-2 | KR073541 | Yam-2 |  |
| 47 | B/Malaysia/U1250/2012 | 24 | Victoria | KR073373 | Vic-1 (Vic-1A) | KR073542 | Vic-1 (Vic-1A) |  |
| 48 | B/Malaysia/U1264/2012 | 59 | Yamagata | KR073374 | Yam-3 (Wisconsin/01) | KR073543 | Yam-3 (Wisconsin/01) |  |
| 49 | B/Malaysia/U1267/2012 | 33 | Victoria | KR073375 | Vic-1 (Vic-1A) | KR073544 | Vic-1 (Vic-1A) |  |
| 50 | B/Malaysia/U1270/2012 | 57 | Yamagata | KR073376 | Yam-2 | KR073545 | Yam-2 |  |
| 51 | B/Malaysia/U1277/2012 | 71 | Victoria | KR073377 | Vic-1 (Vic-1A) | KR073546 | Vic-1 (Vic-1A) |  |
| 52 | B/Malaysia/U1331/2012 | 58 | Victoria | KR073378 | Vic-1 (Vic-1A) | KR073547 | Vic-1 (Vic-1A) |  |
| 53 | B/Malaysia/U1338/2012 | 14 | Yamagata | KR073379 | Yam-3 (Wisconsin/01) | KR073548 | Yam-3 (Wisconsin/01) |  |
| 54 | B/Malaysia/U1429/2012 | 34 | Victoria | KR073380 | Vic-1 (Vic-1B) | KR073549 | Vic-1 (Vic-1A) |  |
| 55 | B/Malaysia/U1463/2012 | 64 | Yamagata | KR073381 | Yam-2 | KR073550 | Yam-2 |  |
| 56 | B/Malaysia/U1531/2012 | 31 | Victoria | KR073382 | Vic-1 (Vic-1A) | KR073551 | Vic-1 (Vic-1A) |  |
| 57 | B/Malaysia/U1573/2012 | 54 | Yamagata | KR073383 | Yam-2 | KR073552 | Yam-2 |  |
| 58 | B/Malaysia/U1580/2012 | 34 | Yamagata | KR073384 | Yam-3 (Stockholm/12) | KR073553 | Yam-3 (Stockholm/12) |  |
| 59 | B/Malaysia/U1593/2012 | 23 | Victoria | KR073385 | Vic-1 (Vic-1A) | KR073554 | Vic-1 (Vic-1A) |  |
| 60 | B/Malaysia/U1710/2012 | 15 | Victoria | KR073386 | Vic-1 (Vic-1A) | KR073555 | Vic-1 (Vic-1A) |  |
| 61 | B/Malaysia/U1725/2012 | 22 | Yamagata | KR073387 | Yam-2 | KR073556 | Yam-2 |  |
| 62 | B/Malaysia/U1827/2013 | 61 | Victoria | KR073388 | Vic-1 (Vic-1A) | KR073557 | Vic-1 (Vic-1A) |  |
| 63 | B/Malaysia/U1846/2013 | 21 | Victoria | KR073389 | Vic-1 (Vic-1A) | KR073558 | Vic-1 (Vic-1A) |  |
| 64 | B/Malaysia/U1876/2013 | 27 | Victoria | KR073390 | Vic-1 (Vic-1A) | KR073559 | Vic-1 (Vic-1A) |  |
| 65 | B/Malaysia/U1879/2013 | 35 | Yamagata | KR073391 | Yam-3 (Wisconsin/01) | KR073560 | Yam-3 (Wisconsin/01) |  |
| 66 | B/Malaysia/U1881/2013 | 58 | Yamagata | KR073392 | Yam-2 | KR073561 | Yam-2 |  |
| 67 | B/Malaysia/U1889/2013 | 71 | Victoria | KR073393 | Vic-1 (Vic-1A) | KR073562 | Vic-1 (Vic-1A) |  |
| 68 | B/Malaysia/U1890/2013 | 27 | Victoria | KR073394 | Vic-1 (Vic-1A) | KR073563 | Vic-1 (Vic-1A) |  |
| 69 | B/Malaysia/U1900/2013 | 62 | Yamagata | KR073395 | Yam-2 | KR073564 | Yam-2 |  |
| 70 | B/Malaysia/U1936/2013 | 57 | Yamagata | KR073396 | Yam-3 (Wisconsin/01) | KR073565 | Yam-3 (Wisconsin/01) |  |
| 71 | B/Malaysia/U1962/2013 | 37 | Yamagata | KR073397 | Yam-2 | KR073566 | Yam-2 |  |
| 72 | B/Malaysia/U1995/2013 | 28 | Yamagata | KR073398 | Yam-3 (Wisconsin/01) | KR073567 | Yam-3 (Wisconsin/01) |  |
| 73 | B/Malaysia/U1996/2013 | 30 | Victoria | KR073399 | Vic-1 (Vic-1A) | KR073568 | Vic-1 (Vic-1A) |  |
| 74 | B/Malaysia/U2002/2013 | 39 | Yamagata | KR073400 | Yam-3 (Wisconsin/01) | KR073569 | Yam-3 (Wisconsin/01) |  |
| 75 | B/Malaysia/U2023/2013 | 32 | Yamagata | KR073401 | Yam-2 | KR073570 | Yam-2 |  |
| 76 | B/Malaysia/U2036/2013 | 15 | Yamagata | KR073402 | Yam-2 | KR073571 | Yam-2 |  |
| 77 | B/Malaysia/U2043/2013 | 56 | Yamagata | KR073403 | Yam-2 | KR073572 | Yam-2 |  |
| 78 | B/Malaysia/U2046/2013 | 71 | Yamagata | KR073404 | Yam-3 (Stockholm/12) | KR073573 | Yam-3 (Stockholm/12) |  |
| 79 | B/Malaysia/U2057/2013 | 24 | Victoria | KR073405 | Vic-1 (Vic-1A) | KR073574 | Vic-1 (Vic-1A) |  |
| 80 | B/Malaysia/U2068/2013 | 41 | Yamagata | KR073406 | Yam-2 | KR073575 | Yam-2 |  |
| 81 | B/Malaysia/U2077/2013 | 33 | Yamagata | KR073407 | Yam-2 | KR073576 | Yam-2 |  |
| 82 | B/Malaysia/U2080/2013 | 47 | Yamagata | KR073408 | Yam-3 (Wisconsin/01) | KR073577 | Yam-3 (Wisconsin/01) |  |
| 83 | B/Malaysia/U2094/2013 | 37 | Yamagata | KR073409 | Yam-3 (Wisconsin/01) | KR073578 | Yam-3 (Wisconsin/01) |  |
| 84 | B/Malaysia/U2111/2013 | 20 | Yamagata | KR073410 | Yam-3 (Wisconsin/01) | KR073579 | Yam-3 (Wisconsin/01) |  |
| 85 | B/Malaysia/U2120/2013 | 25 | Yamagata | KR073411 | Yam-3 (Wisconsin/01) | KR073580 | Yam-3 (Wisconsin/01) |  |
| 86 | B/Malaysia/U2140/2013 | 56 | Yamagata | KR073412 | Yam-2 | KR073581 | Yam-2 |  |
| 87 | B/Malaysia/U2154/2013 | 65 | Yamagata | KR073413 | Yam-3 (Wisconsin/01) | KR073582 | Yam-3 (Wisconsin/01) |  |
| 88 | B/Malaysia/U2163/2013 | 69 | Yamagata | KR073414 | Yam-2 | KR073583 | Yam-2 |  |
| 89 | B/Malaysia/U2177/2013 | 70 | Yamagata | KR073415 | Yam-2 | KR073584 | Yam-2 |  |
| 90 | B/Malaysia/U2179/2013 | 21 | Yamagata | KR073416 | Yam-3 (Wisconsin/01) | KR073585 | Yam-3 (Wisconsin/01) |  |
| 91 | B/Malaysia/U2180/2013 | 33 | Yamagata | KR073417 | Yam-2 | KR073586 | Yam-2 |  |
| 92 | B/Malaysia/U2187/2013 | 15 | Yamagata | KR073418 | Yam-2 | KR073587 | Yam-2 |  |
| 93 | B/Malaysia/U2188/2013 | 55 | Yamagata | KR073419 | Yam-2 | KR073588 | Yam-2 |  |
| 94 | B/Malaysia/U2190/2013 | 71 | Yamagata | KR073420 | Yam-2 | KR073589 | Yam-2 |  |
| 95 | B/Malaysia/U2214/2013 | 28 | Yamagata | KR073421 | Yam-2 | KR073590 | Yam-3 (Stockholm/12) |  |
| 96 | B/Malaysia/U2215/2013 | 32 | Yamagata | KR073422 | Yam-2 | KR073591 | Yam-2 |  |
| 97 | B/Malaysia/U2230/2013 | 20 | Victoria | KR073423 | Vic-1 (Vic-1A) | KR073592 | Vic-1 (Vic-1A) |  |
| 98 | B/Malaysia/U2234/2013 | 78 | Yamagata | KR073424 | Yam-3 (Wisconsin/01) | KR073593 | Yam-3 (Wisconsin/01) |  |
| 99 | B/Malaysia/U2260/2013 | 26 | Yamagata | KR073425 | Yam-2 | KR073594 | Yam-2 |  |
| 100 | B/Malaysia/U2265/2013 | 58 | Yamagata | KR073426 | Yam-3 (Wisconsin/01) | KR073595 | Yam-3 (Wisconsin/01) |  |
| 101 | B/Malaysia/U2292/2013 | 79 | Yamagata | KR073427 | Yam-2 | KR073596 | Yam-2 |  |
| 102 | B/Malaysia/U2299/2013 | 21 | Victoria | KR073428 | Vic-1 (Vic-1A) | KR073597 | Vic-1 (Vic-1A) |  |
| 103 | B/Malaysia/U2305/2013 | 26 | Victoria | KR073429 | Vic-1 (Vic-1A) | KR073598 | Vic-1 (Vic-1A) |  |
| 104 | B/Malaysia/U2335/2013 | 14 | Yamagata | KR073430 | Yam-2 | KR073599 | Yam-2 |  |
| 105 | B/Malaysia/U2343/2013 | 31 | Victoria | KR073431 | Vic-1 (Vic-1A) | KR073600 | Vic-1 (Vic-1A) |  |
| 106 | B/Malaysia/U2363/2013 | 62 | Yamagata | KR073432 | Yam-2 | KR073601 | Yam-2 |  |
| 107 | B/Malaysia/U2368/2013 | 17 | Yamagata | KR073433 | Yam-2 | KR073602 | Yam-2 |  |
| 108 | B/Malaysia/U2370/2013 | 67 | Yamagata | KR073434 | Yam-2 | KR073603 | Yam-2 |  |
| 109 | B/Malaysia/U2388/2013 | 30 | Yamagata | KR073435 | Yam-2 | KR073604 | Yam-2 |  |
| 110 | B/Malaysia/U2396/2013 | 47 | Yamagata | KR073436 | Yam-2 | KR073605 | Yam-2 |  |
| 111 | B/Malaysia/U2409/2013 | 48 | Yamagata | KR073437 | Yam-2 | KR073606 | Yam-2 |  |
| 112 | B/Malaysia/U2425/2013 | 37 | Yamagata | KR073438 | Yam-2 | KR073607 | Yam-2 |  |
| 113 | B/Malaysia/U2447/2013 | 73 | Yamagata | KR073440 | Yam-3 (Wisconsin/01) | KR073608 | Yam-3 (Wisconsin/01) |  |
| 114 | B/Malaysia/U2462/2013 | 26 | Yamagata | KR073441 | Yam-3 (Wisconsin/01) | KR073609 | Yam-3 (Stockholm/12) |  |
| 115 | B/Malaysia/U2501/2013 | 26 | Yamagata | KR073442 | Yam-2 | KR073610 | Yam-2 |  |
| 116 | B/Malaysia/U2527/2013 | 63 | Yamagata | KR073443 | Yam-2 | KR073611 | Yam-2 |  |
| 117 | B/Malaysia/U2542/2013 | 19 | Victoria | KR073444 | Vic-1 (Vic-1A) | KR073612 | Vic-1 (Vic-1A) |  |
| 118 | B/Malaysia/U2547/2013 | 78 | Yamagata | KR073445 | Yam-3 (Wisconsin/01) | KR073613 | Yam-3 (Wisconsin/01) |  |
| 119 | B/Malaysia/U2555/2013 | 21 | Yamagata | KR073446 | Yam-3 (Wisconsin/01) | KR073614 | Yam-3 (Stockholm/12) |  |
| 120 | B/Malaysia/U2782/2013 | 17 | Victoria | KR073447 | Vic-1 (Vic-1A) | KR073615 | Vic-1 (Vic-1A) |  |
| 121 | B/Malaysia/U2807/2013 | 70 | Yamagata | KR073448 | Yam-2 | KR073616 | Yam-2 |  |
| 122 | B/Malaysia/U3224/2013 | 61 | Yamagata | KR073450 | Yam-3 (Wisconsin/01) | KR073617 | Yam-3 (Stockholm/12) |  |
| 123 | B/Malaysia/U3225/2013 | 51 | Yamagata | KR073451 | Yam-3 (Wisconsin/01) | KR073618 | Yam-3 (Stockholm/12) |  |
| 124 | B/Malaysia/U3226/2013 | 20 | Yamagata | KR073452 | Yam-3 (Wisconsin/01) | KR073619 | Yam-3 (Stockholm/12) |  |
| 125 | B/Malaysia/U3244/2013 | 21 | Yamagata | KR073453 | Yam-2 | KR073620 | Yam-2 |  |
| 126 | B/Malaysia/U3261/2013 | 45 | Yamagata | KR073454 | Yam-2 | KR073621 | Yam-2 |  |
| 127 | B/Malaysia/U3277/2013 | 25 | Yamagata | KR073455 | Yam-3 (Wisconsin/01) | KR073622 | Yam-3 (Stockholm/12) |  |
| 128 | B/Malaysia/U3288/2013 | 52 | Yamagata | KR073456 | Yam-3 (Wisconsin/01) | KR073623 | Yam-3 (Stockholm/12) |  |
| 129 | B/Malaysia/U3331/2014 | 27 | Yamagata | KR073457 | Yam-3 (Wisconsin/01) | KR073624 | Yam-3 (Stockholm/12) |  |
| 130 | B/Malaysia/U3328/2014 | 27 | Victoria | KR073458 | Vic-1 (Vic-1A) | KR073625 | Vic-1 (Vic-1A) |  |
| 131 | B/Malaysia/U3340/2014 | 67 | Yamagata | KR073459 | Yam-2 | KR073626 | Yam-2 |  |
| 132 | B/Malaysia/U3349/2014 | 67 | Yamagata | KR073460 | Yam-2 | KR073627 | Yam-2 |  |
| 133 | B/Malaysia/U3404/2014 | 52 | Yamagata | KR073461 | Yam-3 (Wisconsin/01) | KR073628 | Yam-3 (Stockholm/12) |  |
| 134 | B/Malaysia/U3411/2014 | 62 | Yamagata | KR073462 | Yam-3 (Wisconsin/01) | KR073629 | Yam-3 (Stockholm/12) |  |
| 135 | B/Malaysia/U3419/2014 | 49 | Yamagata | KR073463 | Yam-2 | KR073630 | Yam-2 |  |
| 136 | B/Malaysia/U3435/2014 | 34 | Yamagata | KR073464 | Yam-3 (Wisconsin/01) | KR073631 | Yam-3 (Stockholm/12) |  |
| 137 | B/Malaysia/U3488/2014 | 32 | Yamagata | KR073465 | Yam-3 (Wisconsin/01) | KR073632 | Yam-3 (Stockholm/12) |  |
| 138 | B/Malaysia/U3490/2014 | 64 | Yamagata | KR073466 | Yam-2 | KR073633 | Yam-2 |  |
| 139 | B/Malaysia/U3497/2014 | 29 | Yamagata | KR073467 | Yam-3 (Wisconsin/01) | KR073634 | Yam-3 (Stockholm/12) |  |
| 140 | B/Malaysia/U3503/2014 | 19 | Victoria | KR073468 | Vic-1 (Vic-1A) | KR073635 | Vic-1 (Vic-1A) |  |
| 141 | B/Malaysia/U3510/2014 | 28 | Victoria | KR073469 | Vic-1 (Vic-1A) | KR073636 | Vic-1 (Vic-1A) |  |
| 142 | B/Malaysia/U3519/2014 | 61 | Yamagata | KR073470 | Yam-2 | KR073637 | Yam-2 |  |
| 143 | B/Malaysia/U3523/2014 | 21 | Yamagata | KR073471 | Yam-2 | KR073638 | Yam-2 |  |
| 144 | B/Malaysia/U3527/2014 | 54 | Victoria | KR073472 | Vic-1 (Vic-1A) | KR073639 | Vic-1 (Vic-1A) |  |
| 145 | B/Malaysia/U3561/2014 | 60 | Yamagata | KR073473 | Yam-2 | KR073640 | Yam-2 |  |
| 146 | B/Malaysia/U3587/2014 | 26 | Victoria | KR073474 | Vic-1 (Vic-1A) | KR073641 | Vic-1 (Vic-1A) |  |
| 147 | B/Malaysia/U3601/2014 | 46 | Yamagata | KR073475 | Yam-2 | KR073642 | Yam-2 |  |
| 148 | B/Malaysia/U3626/2014 | 49 | Yamagata | KR073476 | Yam-3 (Wisconsin/01) | KR073643 | Yam-3 (Stockholm/12) |  |
| 149 | B/Malaysia/U3630/2014 | 47 | Yamagata | KR073477 | Yam-3 (Wisconsin/01) | KR073644 | Yam-3 (Stockholm/12) |  |
| 150 | B/Malaysia/U3636/2014 | 25 | Yamagata | KR073478 | Yam-3 (Wisconsin/01) | KR073645 | Yam-3 (Stockholm/12) |  |
| 151 | B/Malaysia/U3655/2014 | 52 | Yamagata | KR073479 | Yam-3 (Wisconsin/01) | KR073646 | Yam-3 (Stockholm/12) |  |
| 152 | B/Malaysia/U3656/2014 | 14 | Yamagata | KR073480 | Yam-3 (Wisconsin/01) | KR073647 | Yam-3 (Stockholm/12) |  |
| 153 | B/Malaysia/U3663/2014 | 27 | Yamagata | KR073481 | Yam-3 (Wisconsin/01) | KR073648 | Yam-3 (Stockholm/12) |  |
| 154 | B/Malaysia/U3679/2014 | 45 | Yamagata | KR073482 | Yam-3 (Wisconsin/01) | KR073649 | Yam-3 (Stockholm/12) |  |
| 155 | B/Malaysia/U3685/2014 | 35 | Yamagata | KR073483 | Yam-3 (Wisconsin/01) | KR073650 | Yam-3 (Stockholm/12) |  |
| 156 | B/Malaysia/U3734/2014 | 48 | Yamagata | KR073484 | Yam-3 (Wisconsin/01) | KR073651 | Yam-3 (Stockholm/12) |  |
| 157 | B/Malaysia/U3794/2014 | 61 | Yamagata | KR073485 | Yam-3 (Wisconsin/01) | KR073652 | Yam-3 (Stockholm/12) |  |
| 158 | B/Malaysia/U3802/2014 | 49 | Yamagata | KR073486 | Yam-3 (Wisconsin/01) | KR073653 | Yam-3 (Stockholm/12) |  |
| 159 | B/Malaysia/U3804/2014 | 41 | Yamagata | KR073487 | Yam-2 | KR073654 | Yam-2 |  |
| 160 | B/Malaysia/U3805/2014 | 22 | Yamagata | KR073488 | Yam-3 (Wisconsin/01) | KR073655 | Yam-3 (Stockholm/12) |  |
| 161 | B/Malaysia/U3867/2014 | 43 | Yamagata | KR073490 | Yam-2 | KR073656 | Yam-2 |  |
| 162 | B/Malaysia/U3876/2014 | 23 | Yamagata | KR073491 | Yam-3 (Wisconsin/01) | KR073657 | Yam-3 (Stockholm/12) |  |
| 163 | B/Malaysia/U3895/2014 | 30 | Yamagata | KR073492 | Yam-3 (Wisconsin/01) | KR073658 | Yam-3 (Stockholm/12) |  |
| 164 | B/Malaysia/U3901/2014 | 53 | Yamagata | KR073493 | Yam-3 (Wisconsin/01) | KR073659 | Yam-3 (Stockholm/12) |  |
| 165 | B/Malaysia/9012150273/2009 | 0.7 | Victoria | KX269900 | Vic-5 | KX269937 | Vic-5 | This Study |
| 166 | B/Malaysia/2886678/2010 | 2.5 | Victoria | KX269901 | Vic-1 (Vic-1B) | KX269938 | Vic-1 (Vic-1B) |  |
| 167 | B/Malaysia/2931299/2010 | 10.4 | Victoria | KX269902 | Vic-1 (Vic-1B) | KX269939 | Vic-1 (Vic-1B) |  |
| 168 | B/Malaysia/3023819/2010 | 3.9 | Victoria | KX269903 | Vic-1 (Vic-1A) | KX269940 | Vic-1 (Vic-1A) |  |
| 169 | B/Malaysia/3101843/2010 | 2.3 | Victoria | KX269904 | Vic-1 (Vic-1A) | KX269941 | Vic-1 (Vic-1A) |  |
| 170 | B/Malaysia/3123469/2010 | 1.9 | Victoria | KX269905 | Vic-1 (Vic-1B) | KX269942 | Vic-1 (Vic-1B) |  |
| 171 | B/Malaysia/1052352253/2011 | 2.4 | Victoria | KX269906 | Vic-1 (Vic-1A) | KX269943 | Vic-1 (Vic-1A) |  |
| 172 | B/Malaysia/1102435915/2011 | 12.4 | Victoria | KX269907 | Vic-1 (Vic-1A) | KX269944 | Vic-1 (Vic-1A) |  |
| 173 | B/Malaysia/1122461697/2011 | 11.2 | Victoria | KX269908 | Vic-1 (Vic-1A) | KX269945 | Vic-1 (Vic-1A) |  |
| 174 | B/Malaysia/2022480706/2012 | 5.6 | Victoria | KX269909 | Vic-1 (Vic-1A) | KX269946 | Vic-1 (Vic-1A) |  |
| 175 | B/Malaysia/3020112906/2013 | 2.6 | Yamagata | KX269910 | Yam-2 | KX269947 | Yam-2 |  |
| 176 | B/Malaysia/3030138318/2013 | 0.6 | Yamagata | KX269911 | Yam-3 (Stockholm/12) | KX269948 | Yam-3 (Stockholm/12) |  |
| 177 | B/Malaysia/3040139890/2013 | 15.7 | Victoria | KX269912 | Vic-1 (Vic-1A) | KX269949 | Vic-1 (Vic-1A) |  |
| 178 | B/Malaysia/3040141144/2013 | 1.6 | Victoria | KX269913 | Vic-1 (Vic-1A) | KX269950 | Vic-1 (Vic-1A) |  |
| 179 | B/Malaysia/3040148324/2013 | 2.8 | Victoria | KX269914 | Vic-1 (Vic-1A) | KX269951 | Vic-1 (Vic-1A) |  |
| 180 | B/Malaysia/3050165375/2013 | 5.4 | Yamagata | KX269915 | Yam-2 | KX269952 | Yam-2 |  |
| 181 | B/Malaysia/3070223330/2013 | 2.8 | Yamagata | KX269916 | Yam-2 | KX269953 | Yam-2 |  |
| 182 | B/Malaysia/3110299315/2013 | 2.1 | Victoria | KX269917 | Vic-1 (Vic-1A) | KX269954 | Vic-1 (Vic-1A) |  |
| 183 | B/Malaysia/3120318384/2013 | 2.0 | Yamagata | KX269918 | Yam-3 (Wisconsin/01) | KX269955 | Yam-3 (Stockholm/12) |  |
| 184 | B/Malaysia/3120318925/2013 | 5.3 | Yamagata | KX269919 | Yam-2 | KX269956 | Yam-2 |  |
| 185 | B/Malaysia/4010009302/2014 | 4.2 | Yamagata | KX269920 | Yam-2 | KX269957 | Yam-2 |  |
| 186 | B/Malaysia/4010014621/2014 | 1.0 | Yamagata | KX269921 | Yam-2 | KX269958 | Yam-2 |  |
| 187 | B/Malaysia/4010016227/2014 | 8.9 | Victoria | KX269922 | Vic-1 (Vic-1A) | KX269959 | Vic-1 (Vic-1A) |  |
| 188 | B/Malaysia/4010020385/2014 | 8.4 | Yamagata | KX269923 | Yam-2 | KX269960 | Yam-2 |  |
| 189 | B/Malaysia/4010021941/2014 | 8.4 | Yamagata | KX269924 | Yam-2 | KX269961 | Yam-2 |  |
| 190 | B/Malaysia/4020025624/2014 | 1.0 | Yamagata | KX269925 | Yam-2 | KX269962 | Yam-2 |  |
| 191 | B/Malaysia/4040076207/2014 | 7.9 | Yamagata | KX269926 | Yam-2 | KX269963 | Yam-2 |  |
| 192 | B/Malaysia/4060118607/2014 | 6.0 | Yamagata | KX269927 | Yam-3 (Wisconsin/01) | KX269964 | Yam-3 (Stockholm/12) |  |
| 193 | B/Malaysia/4050106904/2014 | 0.5 | Yamagata | KX269928 | Yam-2 | KX269965 | Yam-2 |  |
| 194 | B/Malaysia/4060119196/2014 | 0.5 | Yamagata | KX269929 | Yam-3 (Wisconsin/01) | KX269966 | Yam-3 (Stockholm/12) |  |
| 195 | B/Malaysia/4070165231/2014 | 4.0 | Yamagata | KX269930 | Yam-3 (Wisconsin/01) | KX269967 | Yam-3 (Stockholm/12) |  |
| 196 | B/Malaysia/4080177060/2014 | 3.9 | Victoria | KX269931 | Vic-1 (Vic-1A) | KX269968 | Vic-1 (Vic-1A) |  |
| 197 | B/Malaysia/5030435227/2015 | 0.9 | Yamagata | KX269932 | Yam-3 (Wisconsin/01) | KX269969 | Yam-3 (Stockholm/12) |  |
| 198 | B/Malaysia/5051020629/2015 | 0.3 | Yamagata | KX269933 | Yam-3 (Wisconsin/01) | KX269970 | Yam-3 (Stockholm/12) |  |
| 199 | B/Malaysia/5051034215/2015 | 0.7 | Victoria | KX269934 | Vic-1 (Vic-1A) | KX269971 | Vic-1 (Vic-1A) |  |
| 200 | B/Malaysia/5051039440/2015 | 6.3 | Yamagata | KX269935 | Yam-3 (Wisconsin/01) | KX269972 | Yam-3 (Stockholm/12) |  |
| 201 | B/Malaysia/5061049976/2015 | 2.3 | Yamagata | KX269936 | Yam-3 (Wisconsin/01) | KX269973 | Yam-3 (Stockholm/12) |  |

**Reference**

1. Oong XY, Ng KT, Lam TT, Pang YK, Chan KG, Hanafi NS, et al. Epidemiological and evolutionary dynamics of influenza B viruses in Malaysia, 2012-2014. PLoS One. 2015;10(8):e0136254. doi:10.1371/journal.pone.0136254
